# Recommendations of the Brazilian Society of Nephrology for the management of chronic kidney disease in primary health care

**DOI:** 10.1590/2175-8239-JBN-2026-0093en

**Published:** 2026-10-02

**Authors:** Farid Samaan, Viviane Calice-Silva, Felipe Costa Neves, Pablo Rodrigues Costa Alves, Geraldo Bezerra da Silva, Lilian Pires de Freitas do Carmo, Verônica de Jesus Rodrigues Cardozo Costa, Máira de Faria Polcheira, René Scalet dos Santos, Patrícia Ferreira Abreu, Gianna Mastroianni Kirsztajn, José A. Moura-Neto

**Affiliations:** 1 Sociedade Brasileira de Nefrologia, São Paulo, SP, Brazil.; 2 Secretaria de Estado da Saúde de São Paulo, São Paulo, SP, Brazil.; 3 Instituto Dante Pazzanese de Cardiologia, São Paulo, SP, Brazil.; 4 Fundação Pró-Rim, Joinville, SC, Brazil.; 5 Escola Bahiana de Medicina e Saúde Pública, Salvador, BA, Brazil.; 6 Universidade Federal da Paraíba, João Pessoa, PB, Brazil.; 7 Universidade de Fortaleza, Fortaleza, CE, Brazil.; 8 Instituto Federal de Educação, Ciência e Tecnologia do Ceará, Fortaleza, CE, Brazil.; 9 Universidade Federal de Minas Gerais, Belo Horizonte, MG, Brazil.; 10 Hospital Evangélico de Belo Horizonte, Belo Horizonte, MG, Brazil.; 11 Secretaria Municipal de Saúde de Parauapebas, Parauapebas, PA, Brazil.; 12 Secretaria de Estado de Saúde do Distrito Federal, Brasília, DF, Brazil.; 13 Chronos Pesquisa Clínica, Taguatinga, DF, Brazil.; 14 Secretaria Municipal de Saúde de Curitiba, Curitiba, PR, Brazil.; 15 Universidade Federal de São Paulo, São Paulo, SP, Brazil.

**Keywords:** Primary Health Care, Screening Programs, Continuing Education, Ambulatory Care, Chronic Kidney Disease, Unified Health System, Consensus

## Abstract

Brazil has been undergoing rapid epidemiological and demographic transition and faces major challenges in addressing chronic noncommunicable diseases. Among these, chronic kidney disease (CKD) stands out, given its growing prevalence and contribution to 3–7% of healthcare spending in many countries. CKD is considered one of the diseases with the greatest potential for the application of preventive measures, as it has common risk factors, low-cost and widely available diagnostic tests, a known natural history, feasible treatment, and proven cost-effectiveness of interventions. Despite many difficulties, Brazil is internationally recognized for having one of the most robust and inclusive public health systems in the world (the Unified Health System–SUS, in Portuguese). One of the reasons for this recognition is the comprehensiveness and multidisciplinary nature of its primary health care (PHC). Although Brazilian and international guidelines have been published and updated for more than two decades, recent studies continue to show gaps in the implementation of the care pathway for people with CKD in Brazil. The objective of this document was to develop practical recommendations to help PHC managers include CKD in their work routine, considering current guidelines, Brazilian laws, financial and structural resources, and the characteristics of the SUS.

## INTRODUCTION

Brazil is an upper-middle-income Latin American country of continental dimensions, currently undergoing a marked epidemiological and demographic transition^1,2^. In recent decades, the percentage of individuals over 60 years of age, in relation to the total population, has increased from 9% to 13%^3^. In the same period, the prevalence of adults with obesity, arterial hypertension (AH), and diabetes mellitus (DM) rose from 11% to 23%, 21% to 25%, and 5% to 9%, respectively^4^.

Like many nations, Brazil faces major challenges in addressing chronic noncommunicable diseases (NCDs)^5,6^. Among these, chronic kidney disease (CKD) stands out, accounting for 3–7% of healthcare spending in many countries^7,8,9^, and its increasing prevalence is closely related to rising life expectancy and the growing prevalence of obesity, AH, and DM^10,11,12^.

CKD is considered one of the diseases with the greatest potential for the application of preventive measures because it has, among other characteristics, common risk factors, low-cost diagnostic tests with wide availability, known natural history, feasible treatment once diagnosed, and proven cost-effectiveness of interventions^13,14,15,16^.

Brazil is a country with a dual health system in which 100% of the population is entitled to public services (including dialysis and kidney transplantation) and around 25% of people have some form of private health insurance^17,18,19^. Even with many difficulties, the Brazilian public health system is internationally recognized for being one of the most robust and inclusive in the world (the Unified Health System, SUS)^20,21^. One of the reasons for this recognition is the comprehensiveness and multidisciplinary nature of the Primary Health Care (PHC) within the SUS^22,23^.

Despite the existence of Brazilian and international CKD guidelines, which have been published and updated for more than two decades^24,25,26,27,28^, recent studies continue to show important gaps in the implementation of the line of care for people with CKD in Brazil and around the world^29,30,31,32,33,34,35^. The objective of this document was to develop practical recommendations to help PHC managers include CKD in their work routine, considering current guidelines, available resources, and the characteristics of the SUS in Brazil.

## METHODS

This document is an institutional position statement of the Brazilian Society of Nephrology (BSN), with an educational and non-regulatory nature.

### Identification of key processes

The first phase in the development of this document was the identification of essential actions that PHC managers must undertake to incorporate CKD into their work routine. For this stage, members of the Executive Board, the Department of Epidemiology and CKD Prevention, and BSN-affiliated physicians were consulted. These individuals had experience in public health management and/or providing matrix support to primary care teams, with at least one representative from each region of Brazil.

### Development of recommendations

Whenever possible, the recommendations were developed based on scientific evidence and Brazilian legislation. Some recommendations were technical in nature, allowing them to be supported by specialized literature and current regulations. Others, however, being essentially managerial in nature, required the development of suggestions based on the authors’ experience.

### Public consultation

The first version of the recommendations was made available for public consultation and disseminated through the Brazilian Society of Nephrology’s website and social media channels. Respondents were invited to complete an electronic form in which they indicated their level of agreement (“agree”, “partially agree”, or “disagree”) and were provided with space to submit comments in a free-text field, with no restriction on the number of suggestions per participant.

## RESULTS

The key processes identified for incorporating CKD into routine PHC work were: (1) federal government responsibilities; (2) responsibilities of states and municipalities; (3) approval of a municipal CKD screening protocol in PHC; (4) standardization of CKD diagnostic test reports; (5) professional training; (6) coordination of comprehensive care across all stages of CKD; and (7) indicator-based management (Chart 1).

**Chart 1:** Key processes for managing CKD in PHC.

Key process	Recommendations
Federal responsibilities	– Formulate public policy to combat CKD in PHC. – Provide financial support to states and municipalities in the implementation of health actions and services. – Keep the SUS Table of Procedures and Medications updated.
State responsibilities	– Provide technical and financial support for the implementation of national CKD policies in municipalities. – Monitor PHC actions in CKD. – Ensure access to specialized nephrology care at the secondary and tertiary levels.
Municipal responsibilities	– Execute healthcare actions and services in PHC. – Prioritize the ESF in the organization of PHC. – Ensure spaces for multidisciplinary and population participation. – Include CKD in the work routine: approval of a municipal protocol, carry out recurring professional training, use performance indicators and targets.
Approval of a CKD protocol in PHC	The CKD protocol in PHC must include at least the following content: – The target population for screening. – The screening tests. – The referral criteria for specialized nephrology care.
Standardization of test reports	– The result of SCr (SUS code: 02.02.01.031-7) must be accompanied by the eGFR. – The result of microalbuminuria (SUS code: 02.02.05.009-2) must be accompanied by the UACR.
Professional training	Professional training must follow the following principles: – Legitimacy: consider the current PNAB, PMS, and PAS and be based on the municipal protocol. – Multidisciplinary approach: doctors, nurses, nutritionists, dentists, pharmacists, social workers, community health agents, among others. – Feasibility and recurrence: training should last 120 minutes and be carried out every six months (suggestion). – Dialogue: training must allow active participation of healthcare professionals directly involved in patient care.
Coordination of comprehensive care in PHC	People referred to secondary and/or tertiary care levels must maintain their link with PHC.
Indicator-based management	Municipal health managers must monitor one or more of the following quality indicators of CKD care in PHC: – % of adults with AH and/or DM and ≥ 1 SCr measurement in 12 months. – % of adults with AH and/or DM and ≥ 1 UACR measurement in 12 months. – % of adults with AH and blood pressure < 130 x 80 mmHg. – % of adults with DM and HbA1c < 7.0%. – % of adults with CKD using ACE inhibitors or ARBs, unless contraindicated. – % of adults with CKD using SGLT2 inhibitors, unless contraindicated.

Abbreviations – CKD: chronic kidney disease; PHC: primary health care; SUS: Unified Health System; SCr: serum creatinine; ESF: Family Health Strategy (*Estratégia Saúde da Família*, in Portuguese); eGFR: estimated glomerular filtration rate; UACR: urinary albumin-to-creatinine ratio in a spot urine sample; PNAB: National Primary Care Policy (*Política Nacional de Atenção Básica*, in Portuguese); PMS: Municipal Health Plan (*Plano Municipal de Saúde*, in Portuguese); PAS: Annual Health Program (*Programação Anual de Saúde*, in Portuguese); AH: arterial hypertension; DM: diabetes mellitus; HbA1c: glycated hemoglobin; ACE inhibitors: angiotensin-converting enzyme inhibitors; ARBs: angiotensin receptor blockers; SGLT2 inhibitors: sodium-glucose cotransporter 2 inhibitors.

### Federal responsibilities

**Recommendation 1**. The Brazilian Ministry of Health (MS, in Portuguese) should establish national guidelines for the management of CKD in PHC and provide financial support to states and municipalities for the implementation of diagnostic and therapeutic procedures, while keeping the reimbursement schedule for service providers up to date. As a common, preventable, and costly condition, CKD should be included in all innovative MS proposals, such as those related to electronic health records within the SUS and performance-based reimbursement (health prevention indicators, provision of integrated care, among others).

**Comment**. The federal government is responsible for establishing the guidelines that govern public health policies. Among the most recent documents related to CKD in Brazil are Ordinance No. 1,675/2018^25^, which sets forth the criteria for organizing care for people with CKD within the SUS, and the 2024 Clinical Protocol and Therapeutic Guidelines (PCDT; *Protocolo Clínico e Diretrizes Terapêuticas*) for CKD^26^, which describes strategies to slow CKD progression. In both Brazilian regulations, the roles of PHC in early diagnosis, treatment of the initial stages of CKD, and coordination of comprehensive patient care are emphasized. Among other recommendations, the CKD PCDT provides specific technical guidance for CKD screening and medication use within the SUS.

### Responsibilities of states and municipalities

**Recommendation 2**. Every municipality in Brazil should have a defined strategy for addressing CKD at the primary care level, regardless of population size. This strategy includes at least two actions: the development of a screening protocol for at-risk populations and the management of low and moderate-risk CKD cases. CKD-related healthcare activities at the secondary and tertiary levels should be agreed upon regionally on a bipartite basis (states and municipalities) and include matrix support, specialized outpatient nephrology care, and kidney replacement therapy (KRT).

**Comment**. According to the National Primary Care Policy (PNAB; *Política Nacional de Atenção Básica*), PHC within the SUS is essentially managed by municipalities, which have autonomy to plan and implement health actions and services^36^. The 2024 CKD PCDT establishes that individuals at risk for CKD should be screened for the disease at the primary care level^26^. In Brazil, approximately 30% of adults have one or more risk factors for CKD—such as hypertension, diabetes mellitus, obesity, and established cardiovascular disease—making PHC essential for the early diagnosis of CKD^4^. When a municipality or region performs the recommended CKD screening tests, it is estimated that more than 97% of individuals will either not have CKD or will have low or moderate-risk CKD according to the disease heat map^32,37,38^ (Table 1). Therefore, municipalities need to be prepared to care for the vast majority of screened patients.

**Table 1:** Percentage distribution of CKD risk categories among adults.

CKD stage	eGFR (mL/min/1.73 m^2^)	UACR level (mg/g)			Total
		Mild < 30	Moderate 30-300	Severe > 300	
1	≥ 90	45.01 (no CKD)	1.99	0.37	47.37
2	60-89	40.72 (no CKD)	1.78	0.31	42.81
3 a	45–59	7.41	0.55	0.13	8.09
3 b	30–44	1.19	0.21	0.07	1.47
4	15–29	0.10	0.05	0.05	0.20
5	< 15	0.03	0.01	0.02	0.06
Total		94.46	4.59	0.95	100.00

Abbreviations – CKD: chronic kidney disease; eGFR: estimated glomerular filtration rate; UACR: urinary albumin-to-creatinine ratio in a spot urine sample.

Notes – Each colored cell in the figure shows the percentage distribution (%) of the corresponding CKD risk category (Ref. 37). CKD risk categories according to KDIGO (Kidney Disease: Improving Global Outcomes) (Ref. 28): green, low risk; yellow, moderately increased risk; orange, high risk; red, very high risk.

In Brazil, 69% of municipalities have fewer than 20,000 inhabitants, a proportion that ranges from 57% to 77% across the country’s five regions (Table 2)^39,40^. Therefore, specialized nephrology care at the secondary and tertiary levels should be guaranteed and organized primarily by the states and the federal government. The current Ministry of Health ordinance on healthcare parameters recommends providing 1,600 nephrology consultations per year for every 100,000 inhabitants^39^.

**Table 2:** Distribution of brazilian municipalities by estimated population in 2025 [40].

Region/UF	Frequency of municipalities by population size, % (n)				
	Fewer than 20,000	20,000 to 49,999	50,000 to 99,999	100,000 to 499,999	500,000 or more	Total
North	57.3 (258)	26.7 (120)	9.6 (43)	5.6 (25)	0.9 (4)	100.0 (450)
AC	63.6 (14)	27.3 (6)	4.5 (1)	4.5 (1)	0.0 (0)	100.0 (22)
AM	35.5 (22)	45.2 (28)	12.9 (8)	4.8 (3)	1.6 (1)	100.0 (62)
AP	68.8 (11)	18.8 (3)	0.0 (0)	12.5 (2)	0.0 (0)	100.0 (16)
PA	27.1 (39)	43.1 (62)	19.4 (28)	9.0 (13)	1.4 (2)	100.0 (144)
RO	65.4 (34)	21.2 (11)	5.8 (3)	5.8 (3)	1.9 (1)	100.0 (52)
RR	53.3 (8)	40.0 (6)	0.0 (0)	6.7 (1)	0.0 (0)	100.0 (15)
TO	93.5 (130)	2.9 (4)	2.2 (3)	1.4 (2)	0.0 (0)	100.0 (139)
Northeast	66.3 (1190)	23.4 (419)	6.5 (116)	3.2 (58)	0.6 (11)	100.0 (1794)
AL	61.7 (63)	28.4 (29)	7.8 (8)	1.0 (1)	1.0 (1)	100.0 (102)
BA	60.4 (252)	28.5 (119)	6.7 (28)	3.8 (16)	0.5 (2)	100.0 (417)
CE	46.7 (86)	32.6 (60)	15.8 (29)	4.3 (8)	0.5 (1)	100.0 (184)
MA	59.0 (128)	30.9 (67)	5.5 (12)	4.1 (9)	0.5 (1)	100.0 (217)
PB	85.2 (190)	9.9 (22)	3.1 (7)	1.3 (3)	0.4 (1)	100.0 (223)
PE	45.9 (85)	35.1 (65)	10.8 (20)	7.0 (13)	1.1 (2)	100.0 (185)
PI	87.9 (197)	9.4 (21)	1.8 (4)	0.4 (1)	0.4 (1)	100.0 (224)
RN	83.2 (139)	10.8 (18)	3.6 (6)	1.8 (3)	0.6 (1)	100.0 (167)
SE	66.7 (50)	24.0 (18)	2.7 (2)	5.3 (4)	1.3 (1)	100.0 (75)
Midwest	72.2 (338)	17.7 (83)	4.3 (20)	4.7 (22)	1.1 (5)	100.0 (468)
DF	0.0 (0)	0.0 (0)	0.0 (0)	0.0 (0)	100.0 (1)	100.0 (1)
GO	76.4 (188)	13.4 (33)	3.3 (8)	6.1 (15)	0.8 (2)	100.0 (246)
MS	55.7 (44)	35.4 (28)	5.1 (4)	2.5 (2)	1.3 (1)	100.0 (79)
MT	74.6 (106)	15.5 (22)	5.6 (8)	3.5 (5)	0.7 (1)	100.0 (142)
Southeast	67.1 (1120)	17.3 (289)	6.2 (103)	8.0 (133)	1.4 (23)	100.0 (1668)
ES	51.3 (40)	33.3 (26)	2.6 (2)	10.3 (8)	2.6 (2)	100.0 (78)
MG	77.7 (663)	13.8 (118)	4.2 (36)	3.8 (32)	0.5 (4)	100.0 (853)
RJ	28.3 (26)	31.5 (29)	8.7 (8)	23.9 (22)	7.6 (7)	100.0 (92)
SP	60.6 (391)	18.0 (116)	8.8 (57)	11.0 (71)	1.6 (10)	100.0 (645)
South	76.7 (913)	13.4 (159)	5.2 (62)	4.4 (52)	0.4 (5)	100.0 (1191)
PR	75.7 (302)	14.8 (59)	3.5 (14)	5.5 (22)	0.5 (2)	100.0 (399)
RS	78.5 (390)	12.3 (61)	5.4 (27)	3.6 (18)	0.2 (1)	100.0 (497)
SC	74.9 (221)	13.2 (39)	7.1 (21)	4.1 (12)	0.7 (2)	100.0 (295)
Total	68.6 (3819)	19.2 (1070)	6.2 (344)	5.2 (290)	0.9 (48)	100.0 (5571)

Abbreviations – UF: federative unit. North Region: AC, Acre; AM, Amazonas; AP, Amapá; RO, Rondônia; RR, Roraima; TO, Tocantins. Northeast Region: AL, Alagoas; BA, Bahia; CE, Ceará; MA, Maranhão; PB, Paraíba; PE, Pernambuco; PI, Piauí; RN, Rio Grande do Norte; SE, Sergipe. Midwest Region: GO, Goiás; DF, Federal District; MS, Mato Grosso do Sul; MT, Mato Grosso. Southeast Region: ES, Espírito Santo; MG, Minas Gerais; RJ, Rio de Janeiro; SP, São Paulo. South Region: PR, Paraná; RS, Rio Grande do Sul; SC, Santa Catarina.

Note – Each cell of the table shows the absolute and relative frequencies of municipalities by population size, as defined by the population categories shown in the columns.

The following trend in relative healthcare spending by the three levels of government in Brazil (municipal, state, and federal) is recognized: increasing municipal participation, reduced federal funding, and stable state spending^41,42^. Many Brazilian municipalities may have difficulty financing simple diagnostic procedures, such as routine tests for people with AH and DM, thus requiring financial support from the state and/or the federal government for this purpose. However, even in these cases, the implementation of actions at the primary care level remains the responsibility of the municipal government^36^.

### Municipal protocol for CKD screening in PHC

Although Brazilian and international guidelines for the management of CKD are available^24,26,27,28^, it is known that the development of internal protocols by municipalities reflects the dissemination and discussion of the topic among healthcare managers and professionals. The development of a municipal protocol allows for adaptations that take into account the economic, cultural, and social diversity, as well as differences in access to healthcare services, across Brazil.

The municipal CKD screening protocol in PHC should guide the activities of healthcare professionals, providing clear information on, at a minimum, the following questions: What is the target population? Who can order the exams? Which tests should be ordered, and at what frequency? When should CKD cases be referred to a nephrologist?

**Recommendation 3**. The target population for CKD screening in PHC should consist of adults with one or more of the following risk factors: AH, DM, obesity, smoking, advanced age (≥ 60 years), established cardiovascular or cerebrovascular disease, a history of CKD in first-degree relatives, chronic use of nephrotoxic medications, autoimmune diseases with systemic involvement, and chronic urinary tract disorders (recurrent infection or stones, prostatic hyperplasia or neoplasia, chronic bladder dysfunction, among others).

**Comment**. The main justifications for individuals, even if asymptomatic, to undergo CKD screening tests when they have one or more risk factors are as follows: the prevalence of CKD in adults is 8–14% worldwide^43^; it is estimated that only 10–20% of people with CKD are aware of their diagnosis^44^; CKD may not present symptoms in its initial stages, mainly because of the kidneys’ ability to adapt to the loss of nephrons^45^; the presence and severity of CKD are strongly related to cardiovascular events, hospitalizations, the need for KRT, death, impairment of quality of life, and healthcare costs^46,47,48^. It should be noted that, according to Brazilian guidelines and international consensus, CKD screening is only indicated for individuals with risk factors for the disease^24,25,26,27,28^. To date, kidney assessment tests are not recommended for people without risk factors for CKD and under the age of 60^49^. Although there is no scientific evidence to support CKD screening in this population, annual serum creatinine and routine urinalysis testing in the general population can be considered low-cost in some contexts and may be useful for identifying cases of CKD of unknown origin.

**Recommendation 4**. Doctors working in PHC should be trained to request CKD screening tests in accordance with current care protocols (eGFR and UACR).

**Comment**. The details of the roles and responsibilities of the different PHC professional categories must be defined at the municipal level, in line with the PNAB and current legislation^36^. The organization of CKD screening and monitoring actions must respect local care pathway, the installed capacity of services, and the coordination of care within the scope of PHC. Experiences described in different regions of the country demonstrate that the structuring of protocolized actions to evaluate prevalent conditions and to screen for chronic diseases, integrated into well-defined care pathway and with medical support, contributes to expanding access to and the effectiveness of PHC^50,51,52,53,54^. CKD screening is justified by the high prevalence of easily identifiable risk factors in PHC, such as high blood pressure and diabetes mellitus, and by the availability of low-cost, widely accessible, and non-invasive tests in the SUS^18^.

**Recommendation 5**. All municipalities should standardize, in their CKD protocols for PHC, the performance of two tests to screen for the disease: serum creatinine (SUS code: 02.02.01.031-7) and the urinary albumin-to-creatinine ratio (UACR) measured in a spot urine sample (SUS codes: measurement of urinary “microalbumin”, 02.02.05.009-2, and urinary creatinine, 02.02.01.031-7).

**Recommendation 6**. Public managers, whether through their own services or contracted providers, must agree with laboratories that: (1) all serum creatinine (SCr) results, performed for individuals over 18 years of age, must be accompanied by the estimated glomerular filtration rate calculated using the 2021 CKD-EPI (Chronic Kidney Disease Epidemiology Collaboration) equation (eGFR, in mL/min/1.73 m^2^); and (2) every urinary albumin (“microalbuminuria”) result must be accompanied by the albumin-to-creatinine ratio (UACR, in mg/g).

**Comment**. Serum creatinine (SUS code: 02.02.01.031-7) and urinary “microalbumin” or microalbuminuria measurement (SUS code: 02.02.05.009-2) are the terms included in the list of diagnostic and therapeutic procedures offered by the SUS; that is, they are standardized in the Management System for the Table of Procedures, Medications, and OPM (Orthoses, Prostheses, and Materials) (SIGTAP, in Portuguese)^18^. Although SCr is the best-known and most widely available marker of kidney function in Brazil and worldwide, this biomarker is influenced, among other factors, by the individual’s muscle mass^55,56^. For this reason, for more than four decades, the scientific community has developed equations to calculate the eGFR from serum creatinine, using variables such as age, sex, race, weight, and others^57,58,59,60^. Studies have shown that automatically including eGFR in serum creatinine reports can increase the identification of the early stages of CKD, increase the use of nephroprotective medications, and reduce the prescription of nephrotoxic drugs^61^. The formula currently recommended for estimating adult GFR from serum creatinine is the 2021 CKD-EPI equation, which requires only age and sex as variables (Figure 1)^26,28,60^.

**Figure 1:**
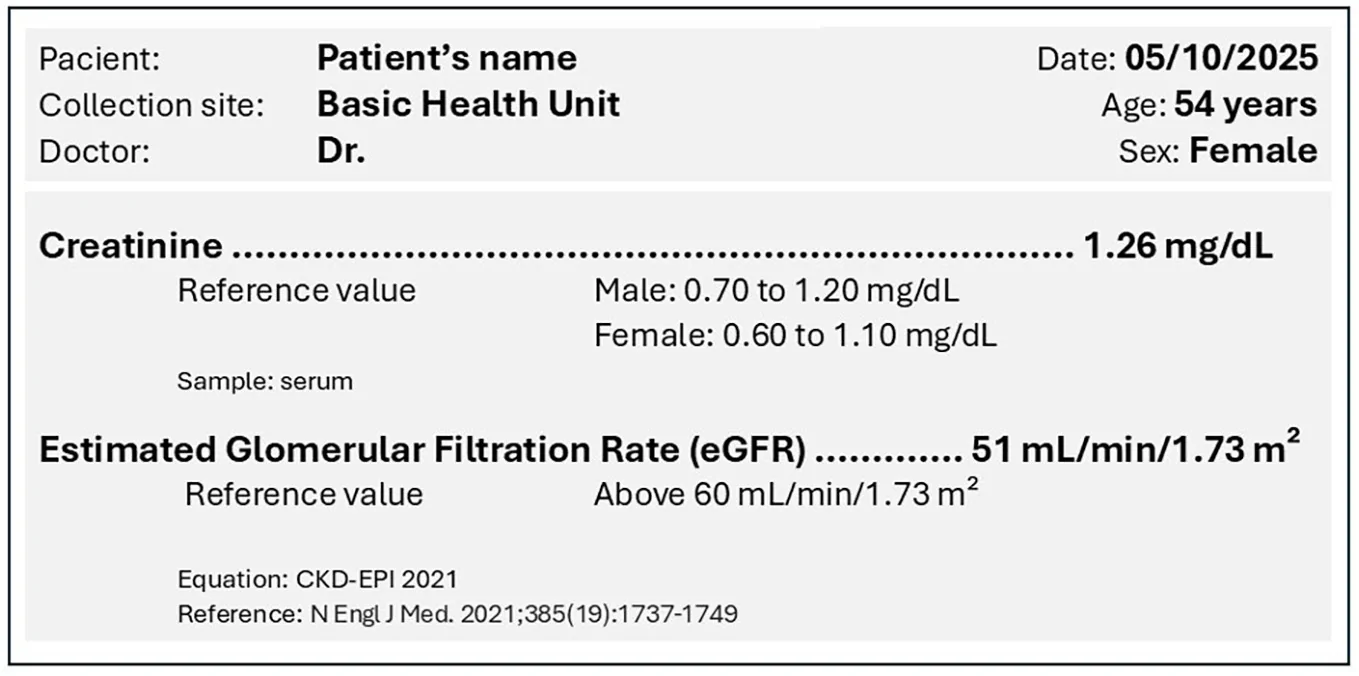
Sample serum creatinine test report (SUS code: 02.02.01.031-7) for individuals aged 18 years or older.

eGFR alone is not sufficient for early diagnosis and risk stratification of CKD^62,63,64^. All public policies to combat CKD must guarantee physical resources and professional training so that a urinary CKD prognostic marker is added to the disease screening routine in addition to eGFR assessment^26,28^. The best marker of CKD in clinical practice is proteinuria, and the most important urinary protein for routine use in PHC is albumin^65,66^. It is recommended that albuminuria, as with other urinary biomarkers, be standardized to urinary creatinine^67^. Therefore, the term “microalbuminuria” is not appropriate, and it is recommended that it be replaced by UACR in the SUS procedure table^28,65,66^. For UACR measurement, laboratories that provide services to the SUS can charge for two codes from the SIGTAP Table: 02.02.05.009-2 (microalbuminuria) and 02.02.01.031-7 (urinary creatinine measurement) (Figure 2). Serum and urinary creatinine levels have the same code in the SIGTAP Table (02.02.01.031-7).

**Figure 2:**
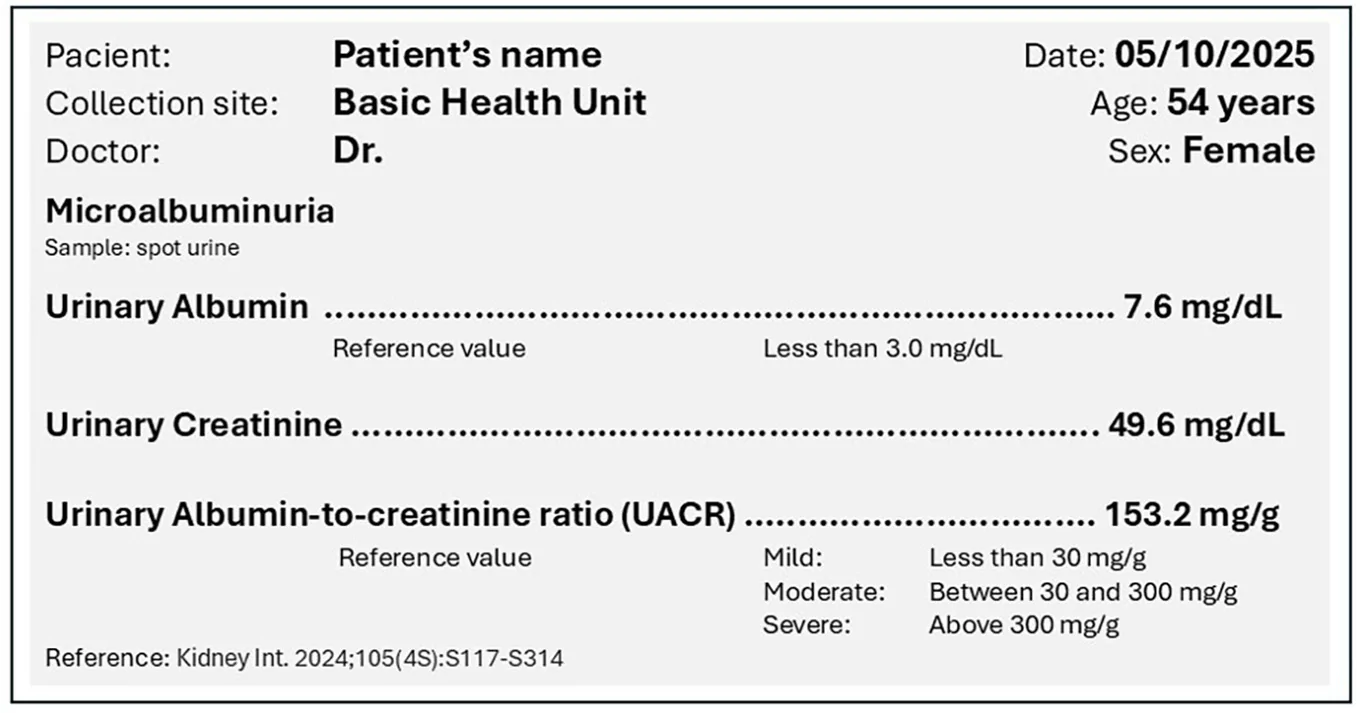
Sample report for the “microalbuminuria” test (SUS codes: 02.02.05.009-2 and 02.02.01.031-7).

There are several methods for evaluating proteinuria, and although there is good equivalence between them, for CKD screening purposes, measurement in an isolated urine sample is preferable to measurement in a 24-hour urine collection^27,28,29^. UACR has a higher cost than routine urinalysis and PCR tests, but its relative cost is low compared to the amount that is routinely spent on clinical laboratory tests for people with AH or DM in the SUS^18,68^. Furthermore, UACR is more sensitive than other methods performed on isolated urine samples for the early diagnosis and prognosis of CKD^69^ (Table 3).

**Table 3:** Equivalence of proteinuria measurements [27,65].

Exam name	Type of protein measurement	Level of proteinuria		
		Mild or absent	Moderate	Severe
Routine urinalysis or urine dipstick	Qualitative	Absent/traces	1+	2+ or 3+
UACR (mg/g)	Quantitative	<30	30–300	>300
UPCR (g/g)	Quantitative	<0,2	0.2–1.0	>1.0
24-hour albuminuria (mg/24 h)	Quantitative	<30	30–300	>300
24-hour proteinuria (mg/24 h)	Quantitative	<150	150–500	>500

Abbreviations – UACR: urinary albumin-to-creatinine ratio; UPCR: urine protein-to-creatinine ratio.

The relevance of eGFR and UACR for routine CKD care in the SUS is reinforced by the need to know the results of these tests not only for diagnosis and risk stratification but also for prescribing and requesting essential nephroprotective medications (angiotensin-converting enzyme inhibitors, angiotensin receptor blockers, and mineralocorticoid receptor antagonists) and the specialized component (type 2 sodium-glucose cotransporter inhibitors)^26,70^ (Chart 2).

**Chart 2:** Nephroprotective medications available in the unified health system (SUS) in 2025 [26,70].

Class	Medicine	Indications for CKD	Contraindications
ACEI	Captopril Enalapril	DM: UACR > 30 mg/g Non-DM: UACR ≥ 300 mg/g	Refractory hyperkalemia Bilateral renal artery stenosis
ARBs	Losartan Valsartan	DM: UACR > 30 mg/g Non-DM: UACR ≥ 300 mg/g	Refractory hyperkalemia Bilateral renal artery stenosis
SGLT2 inhibitors	Dapagliflozin	DM: eGFR 25-75 mL/min/1.73 m^2^ Non-DM: eGFR 25-75 mL/min/1.73 m^2^ and UACR ≥ 300 mg/g	Type 1 DM Patients on dialysis
Mineralocorticoid antagonist	Spironolactone	eGFR > 15 mL/min/1.73 m^2^ and UACR ≥ 300 mg/g	Refractory hyperkalemia

Abbreviations – ACE inhibitors: angiotensin-converting enzyme inhibitors; ARBs: angiotensin receptor blockers; SGLT2 inhibitors: sodium-glucose cotransporter 2 inhibitors; DM: diabetes mellitus; UACR: urinary albumin-to-creatinine ratio; eGFR: estimated glomerular filtration rate.

**Recommendation 7**. Municipalities where people lack access to the conventional laboratory network should consider the use of CKD screening tests carried out at the point of care.

**Comment**. The use of point-of-care tests is endorsed by the current international CKD consensus (Kidney Disease: Improving Global Outcomes [KDIGO], 2024) and by local guidelines^28,71^. It is well established that vulnerable populations with limited access to basic health services, such as clinical laboratory testing, have a higher risk and greater severity of CKD^72,73,74,75^. It should also be highlighted that the benefit and feasibility of point-of-care serum creatinine and proteinuria testing have been demonstrated in different countries, regardless of their national income level^76,77,78,79^. In Brazil, this strategy has also proven feasible. In March 2025, during the World Kidney Day campaign, the Brazilian Society of Nephrology carried out CKD screening activities using serum creatinine and urinary albumin-to-creatinine ratio tests performed at the point of care^80^.

**Recommendation 8**. The recommended minimum number of medical consultations per year for patients with CKD should vary according to the risk stratification of the disease determined using the risk map (heat map): low risk (once per year), moderately increased risk (twice per year), high risk (3 times per year), very high risk (4 times per year; 6–12 times per year if CKD stage 5).

**Comment**. Studies have indicated that the CKD risk map (heat map) has a significant association with renal and non-renal events, including CKD progression, the need for KRT, cardiovascular events (acute myocardial infarction, stroke, arrhythmias, peripheral arterial disease), hospitalization, and death from all causes^81^. Therefore, a progressive increase in the frequency of medical monitoring is justified as the CKD risk category increases. The frequency of consultations should also be adjusted depending on the presence of symptoms and the rate of CKD progression.

**Recommendation 9**. Municipalities should implement routine referral of CKD cases classified as high risk and very high risk according to the CKD heat map for evaluation by a nephrologist.

**Comment**. It is estimated that around 3% of individuals screened with eGFR and UACR fall into the high-risk or very high-risk categories of the CKD heat map (Table 1)^28,37^. This number of people is high given the availability of nephrologists in some regions of the country^82^. For this reason, several guidelines and protocols adopt individuals with CKD stages 4 and 5 as a priority criterion for referral to specialized care, as these patients correspond to approximately 0.3% of the screened population^26,37^. This strategy seeks to reconcile the need for specialized care with the installed capacity of health systems.

However, the current consensus of the Brazilian Society of Nephrology proposes a more comprehensive approach and, whenever possible, an earlier referral, also including individuals with CKD stage 3b and those with severe albuminuria (A3), even in the presence of preserved eGFR. This recommendation is based on consistent evidence that albuminuria and a more pronounced reduction in eGFR are independent markers of worse renal and cardiovascular prognosis and are associated with a greater risk of requiring KRT, cardiovascular events, hospitalizations, and death^83,84,85^. Additionally, it is known that health professionals’ awareness of the importance of CKD screening with UACR assessment is still limited in Brazil and other countries^29,86^. As a consequence, many individuals with UACR ≥ 30 mg/g and preserved eGFR remain unrecognized, delaying the diagnosis and timely treatment of hypertensive and diabetic nephropathies, as well as primary glomerulopathies.

**Recommendation 10**. Monitoring patients with CKD in PHC should include, but not be restricted to: (1) guidance on lifestyle modification; (2) prescription of medications that delay the progression of CKD, when indicated; (3) assessment and treatment of comorbidities and complications of CKD; and (4) avoidance of non-steroidal anti-inflammatory drugs.

**Comment**. Considering Recommendation 9, around 97% of patients screened for CKD should be monitored only in PHC (Table 1). If the municipality opts for a more restrictive routine, that is, recommending referral to a nephrologist only for CKD stages 4 and 5 cases, this percentage will exceed 99%. In both cases, there must be clear protocols for the management of CKD in PHC.

The recommended tests for the management of CKD in PHC should include, but not be restricted to: complete blood count, serum creatinine, routine urinalysis, UACR, fasting blood glucose, glycated hemoglobin (in patients with DM), total cholesterol and fractions, triglycerides, potassium, 25-hydroxyvitamin D, calcium, phosphorus, iron profile (iron, ferritin, and transferrin), parathyroid hormone, and serology for hepatitis B, hepatitis C, and HIV. The suggested time interval for carrying out these tests is described in Table 4; however, it may vary according to the patient’s specific clinical condition^24,26,27,28^. The routine inclusion of serum sodium measurement (given the high percentage of individuals using diuretics) and VDRL (considering the high prevalence of syphilis in the Brazilian population and its association with glomerulopathies) should be considered.

**Table 4:** Recommended tests for monitoring ckd (adapted from refs. 24, 26-28).

CKD stage	Procedures	Interval	Level of care
1 and 2 (eGFR ≥ 60)	– eGFR, routine urinalysis, UACR, and K – Blood count – Blood glucose, HbA1c (if DM) – Total cholesterol and fractions, triglycerides	12 months	Follow up at the PHC Refer to secondary care level if UACR ≥ 300
3 a (eGFR 45-59)	– eGFR, routine urinalysis, UACR, and K – Blood count – Blood glucose, HbA1c (if DM) – Iron, ferritin, transferrin – Total cholesterol and fractions, triglycerides – HBsAg, Anti-HBs, Anti-HBc-total – Anti-HCV, Anti-HIV	12 months	Follow up at the PHC Refer to secondary care level if UACR ≥ 30
3 b (eGFR 30-44)	– eGFR, routine urinalysis, UACR, and K – Blood count – Blood glucose, HbA1c (if DM) – Iron, ferritin, transferrin	6 months	Refer to secondary care level Maintain follow-up at the PHC
	– Total cholesterol and fractions, triglycerides – Ca, P, PTH, 25-hydroxyvitamin D – HBsAg, Anti-HBs, Anti-HBc-total – Anti-HCV, Anti-HIV	12 months	
4 (eGFR 15-29)	– eGFR, routine urinalysis, UACR, and K – Blood count – Blood glucose, HbA1c (if DM) – Iron, ferritin, transferrin	3 months	Refer to secondary care level Maintain follow-up at the PHC
	– Total cholesterol and fractions, triglycerides – Ca, P, PTH, 25-hydroxyvitamin D – Total proteins and fractions	6 months	
	– ECG, Chest X-ray – Ultrasound of kidneys and urinary tract – HBsAg, Anti-HBs, Anti-HBc-total – Anti-HCV, Anti-HIV	12 months
5 (eGFR < 15)	– Hemoglobin, hematocrit – Urea, creatinine, Na, K, Ca, P – Venous blood gas analysis	1 month	Refer to secondary care level Maintain follow-up at the PHC
	– Blood count – Iron, ferritin, transferrin	3 months	
	– Blood glucose, HbA1c (if DM) – Total cholesterol and fractions, triglycerides – Ca, P, PTH, 25-hydroxyvitamin D – Total proteins and fractions	6 months	Refer to secondary care level Maintain follow-up at the PHC
	– ECG, Chest X-ray – Ultrasound of kidneys and urinary tract – HBsAg, Anti-HBs, Anti-HBc-total – Anti-HCV, Anti-HIV	12 months	

Abbreviations – CKD: chronic kidney disease; eGFR: estimated glomerular filtration rate (in mL/min/1.73 m^2^); UACR: urinary albumin-to-creatinine ratio (in mg/g); K: potassium; HbA1c: glycated hemoglobin; DM: diabetes mellitus; Ca: calcium; P: phosphorus; PTH: parathyroid hormone; Na: sodium; ECG: electrocardiogram; PHC: primary health care; HBsAg: hepatitis B surface antigen; Anti-HBs: antibody to hepatitis B surface antigen; Anti-HBc-total: total antibody to hepatitis B core antigen; Anti-HCV: antibody to hepatitis C virus; Anti-HIV: antibody to human immunodeficiency virus (HIV).

### Professional training

**Recommendation 11**. The topic of CKD should be part of the SUS Permanent Health Education (EPS, in Portuguese) programs. The content and format of professional training must be defined jointly by professionals from PHC and secondary nephrology care.

**Comment**. EPS is a SUS strategy regulated by the Ministry of Health and defined in the National Policy for Permanent Health Education (PNEPS, in Portuguese)^87^. Considering the absence of nephrologists in most Brazilian municipalities, states must guarantee technical and educational support to municipalities so that the problem-solving capacity of PHC is maximized. Secondary care is responsible for providing matrix support to PHC^25,26^. EPS activities in the context of CKD must be agreed upon in bipartite technical groups (Bipartite Inter-managerial Commission – CIB, in Portuguese) and regional groups (Regional Inter-managerial Commission – CIR, in Portuguese)^88^.

The justification for CKD training to be developed jointly by nephrologists and PHC professionals is related to the fact that the latter generally have greater experience in comprehensive patient care, greater experience with teamwork, and greater knowledge of patients’ living conditions and the social determinants of health in the territory^89,90^.

**Recommendation 12**. Whenever possible, professional training in CKD should be carried out with multiple PHC professional categories, including, but not restricted to, physicians, nurses, nursing technicians and assistants, dental surgeons, nutritionists, and community health workers (CHWs).

**Comment**. Multidisciplinary and transdisciplinary teamwork is an essential characteristic of the Family Health Strategy (ESF, in Portuguese) and PHC in the SUS^91^. Studies have shown that care models based on the ESF, compared with traditional models of care in PHC, are associated with better indicators of quality of care and greater access to health services, especially for people with greater social vulnerability^92,93,94,95^. The positive impact of the participation of community health workers in PHC on the quality of CKD care has also been demonstrated^96,97^.

**Recommendation 13**. EPS activities must be aligned with the PNAB, current municipal planning actions, and the CKD protocol previously approved by the municipality, and must minimally include the following topics: CKD screening, CKD management in PHC, and referral criteria for secondary care.

**Comment**. Professionals responsible for developing EPS actions must consider the PNAB, work routines, and the autonomy of municipalities in PHC management^36^. Whenever possible, EPS activities on CKD should be legitimized by the Municipal Health Plan and the Annual Health Program and should be included in the annual budget for the corresponding year^98^. The municipal CKD screening protocol, detailed in Section 3.3 of this document (Recommendations 3 through 10), must be approved prior to the start of EPS activities to give legitimacy to the content taught.

**Recommendation 14**. EPS actions on the topic of CKD in PHC should: (1) be carried out during workers’ normal working hours; (2) be recurring and of short duration; (3) have defined objectives and indicators; (4) use ­problem-based learning methodologies; and (5) include synchronous interactions between students and teachers.

**Comment**. The ultimate objective of the PNEPS should be the transformation of healthcare practices through the development of SUS professionals, thus increasing the autonomy and problem-solving capacity of PHC^87^. To achieve these results, EPS actions must consider the following factors: many health workers have more than one employment relationship (especially physicians)^99,100,101^; there is high turnover among SUS workers, a phenomenon that is even more pronounced in PHC^102,103,104^; and, in general, there is a high demand for care at health units.

Therefore, it is essential that educational activities take place during normal working hours to increase adherence by health professionals; be recurring so that the knowledge of new professionals about work routines is aligned with that of more experienced professionals; have pragmatic content, clear objectives, and measurable indicators; and have a duration that takes into account the care demand of the health unit and allow dialogue and the exchange of ideas between teachers and students, since the degree of experience in the local context may vary among the professionals involved.

### Coordination of comprehensive care across all stages of CKD

**Recommendation 15**. The governments of the three federative entities must join forces to guarantee conditions necessary for PHC to coordinate comprehensive care for people with CKD at all stages.

**Comment**. People classified as having high-risk and very high-risk CKD and those on KRT should be managed at the secondary and tertiary levels of care, respectively^25,26,27,28^. Furthermore, these patients must maintain a link with PHC, considering the following specific needs, including but not limited to vaccination, oral health care, dispensing of essential medicines, health transport, and support for accessing social assistance^25,36^.

Compared to the general population, individuals with CKD have a higher average age, a greater burden of disease, and greater social vulnerability and, therefore, greater health demands^105,106,107^. Over the last century, health sciences, as an area of knowledge, have shown an exponential increase in new discoveries^108,109^. Along with this phenomenon, the number of medical and non-medical specialties has increased, as have life expectancy and the prevalence of chronic diseases^110,111^. As a consequence, care for people with multimorbidity has become a challenge for health systems^112,113^. Despite current Brazilian legislation, which is very clear regarding the role of PHC as the level of care responsible for coordinating comprehensive care^25,36^, funding limitations, a lack of technological resources, and the absence of optimal conditions for the development of public health managers—especially at the PHC level^114,115,116,117^—must be acknowledged.

PHC should coordinate a continuum of care that promotes comprehensive care and resolution. Comprehensive care should be guided by longitudinality, ensuring a continuous bond between PHC professionals and users over time, regardless of the stage of CKD^25^. In the case of patients with high-risk and very high-risk CKD, PHC should be the hub for regional communication, thus reducing the risks of iatrogenesis resulting from duplicated interventions and ensuring the delivery of care—whether or not related to CKD^118,119,120^.

### Indicator-based management

**Recommendation 16**. Whenever possible, CKD management actions in PHC should be monitored using quality indicators with the following characteristics: scientific relevance, objectivity, data availability, feasible assessment cost, reproducibility, and defined comparison targets (benchmarks).

**Comment**. The use of indicators is essential in public management, especially in a scenario of rising disease burden, increasing healthcare expenditures, and limited resources^121,122^. The choice of indicator(s) must be made carefully and based on several prerequisites, including: scientific relevance (the indicator must be associated with important outcomes such as complication and death rates); objectivity (the calculation formula must be clearly defined); feasibility (the data necessary to calculate the indicator must be available, and the cost of obtaining these data must be feasible given local conditions); reproducibility (the indicator must be monitored over time at the same location and be capable of being monitored at other locations); and the presence of comparison targets (the indicator must have a target defined based on previously published experiences, benchmarks)^123,124^.

It must be recognized that, in daily practice, especially in low and middle-income countries, obtaining indicators that have all these characteristics is often not possible^125^. In the context of CKD care, there are indicators that reasonably meet the requirements described above^126,127,128,129,130^. Furthermore, these indicators can be used separately for CKD management at the primary and secondary levels (Chart 3).

**Chart 3:** Main quality indicators of CKD care in primary and secondary healthcare levels (adapted from refs. 126–130).

Level of attention	Indicators
Primary	% of people with AH and/or DM and ≥ 1 eGFR test in 12 months. % of people with AH and/or DM and ≥ 1 UACR test in 12 months. % of people with AH and BP < 130 x 80 mmHg. % of people with DM and HbA1c < 7.0%. % of people with CKD using ACE inhibitors or ARBs, unless contraindicated. % of people with CKD using SGLT2 inhibitors, unless contraindicated. % of people with CKD who have the diagnosis recorded in their medical records.
Secondary	% of people with AH and BP < 130 x 80 mmHg. % of people with DM and HbA1c < 7.0%. % of people with CKD using ACE inhibitors or ARBs, unless contraindicated. % of people with CKD using SGLT2 inhibitors, unless contraindicated. % starting HD with a functioning AVF. % starting KRT without hospitalization. % starting KRT on peritoneal dialysis. % referred for preemptive kidney transplantation.

Abbreviations – CKD: chronic kidney disease; AH: arterial hypertension; DM: diabetes mellitus; eGFR: estimated glomerular filtration rate; UACR: urinary albumin-to-creatinine ratio in a spot urine sample; BP: blood pressure; HbA1c: glycated hemoglobin; ACE inhibitors: angiotensin-converting enzyme inhibitors; ARBs: angiotensin receptor blockers; SGLT2 inhibitors: sodium-glucose cotransporter 2 inhibitors; AVF: arteriovenous fistula; KRT: kidney replacement therapy.

The main indicators of the quality of CKD care in PHC are related to eGFR testing in patients at risk for CKD, UACR testing in patients at risk for CKD, control of AH and DM, use of nephroprotective medications unless contraindicated, documentation of the diagnosis of CKD, and referral of patients at high or very high risk of CKD to secondary care level. The technical summary sheets of these indicators, with the proposed goals, are described in Table 5 ^131,132,133,134,135,136,137,138,139^. Defining goals for the selected indicators is often a subjective task, since the physical, human, and financial resources available locally, as well as the individual characteristics of the patients (age, comorbidities, tolerance to available medications, among others), must be considered^140^. In addition to defining these goals, public managers must establish action plans to improve the results of the indicators^140,141^. These plans must clearly and objectively describe the activities to be carried out, the professionals responsible, the implementation deadlines, and the monitoring intervals^141^.

**Table 5:** Technical specifications of the quality indicators selected to monitor CKD care in PHC.

Indicator	Numerator	Denominator	Unit	Target	Variation	Ref.
eGFR testing in people with AH and/or DM	Number of adults with AH and/or DM and ≥ 1 eGFR measurement in 12 months	Number of adults with AH and/or DM	%	90%	Positive*	29, 131, 132
UACR testing in people with AH and/or DM	Number of adults with AH and/or DM and ≥ 1 UACR measurement in 12 months	Number of adults with AH and/or DM	%	50%	Positive	29, 131, 132
AH Control	Number of adults with AH and BP < 130 x 80 mmHg	Number of adults with AH	%	70%	Positive	29, 133
DM Control	Number of adults with DM and HbA1c < 7.0%	Number of adults with DM	%	50%	Positive	29, 134
Use of ACE inhibitors/ARBs, unless contraindicated	Number of adults with CKD using ACE inhibitors or ARBs, unless contraindicated	Number of adults with CKD	%	80%	Positive	29, 131
Use of iSGLT2, unless contraindicated	Number of adults with CKD using SGLT2 inhibitors, unless contraindicated	Number of adults with CKD	%	50%	Positive	30, 135–137
Recognition of CKD diagnosis	Number of adults with a documented diagnosis of CKD in their medical records	Number of adults with CKD	%	40%	Positive	29, 138, 139

Abbreviations – eGFR: estimated glomerular filtration rate; AH: arterial hypertension; DM: diabetes mellitus; ACE inhibitors: angiotensin-converting enzyme inhibitors; ARBs: angiotensin receptor blockers; SGLT2 inhibitors: sodium-glucose cotransporter 2 inhibitors; Ref.: reference.

Note – *Positive variation in the indicator: the higher the result, the better.

## DISCUSSION

This BSN consensus article summarizes a set of 17 recommen­dations to support the management of CKD in PHC within the SUS. All recommendations were supported by Brazilian legislation and specialized scientific literature, including ordinances, national clinical guidelines, and recent international consensus statements. Furthermore, the content was widely discussed by BSN members and managers from the five regions of Brazil, ensuring legitimacy and representativeness.

Despite underfunding and the need to modernize public health management, especially at the primary care level, PHC within the SUS is recognized worldwide for its scope and results in vaccination campaigns, fighting endemic diseases, and other initiatives^142,143,144^. However, the epidemiological and demographic changes that Brazil is undergoing have created challenging health demands^1,6,145,146^. Among them, the NCDs stand out, with CKD being a major contributor. Compared with DM, cardiovascular diseases, chronic respiratory diseases, and malignant neoplasms, CKD is the condition that has shown the greatest increase in prevalence and the greatest impact on reduced life expectancy and healthcare expenditures^147^.

*Per capita* health expenditure in Brazil is low when compared with that of high-income countries. Furthermore, only 50% of these resources are allocated to public healthcare, on which just over 75% of Brazilians depend exclusively^148,149^. Among the levels of government (federal, state, and municipal), municipalities account for the largest share of health expenditures, which consume a large proportion of their annual budgets^41,42,150^.

The ESF is a well-established care model, both because of its better performance on care indicators and because of the higher perception of quality by users when compared with the traditional primary health care model^92,93,94,95,151^. ESF coverage is uneven across the regions of Brazil, and there is also considerable heterogeneity within states^151,152^. Furthermore, the stagnation/reduction in the coverage of community health workers, the professional category with the closest contact with the population, is worrying^153,154^.

The implementation of the recommendations depends on coordination between the SUS governance and management bodies, such as Municipal and State Health Secretariats and the inter-managerial committees (CIR and CIB), which are responsible for defining care pathways and incorporating CKD into intergovernmental health agendas^36,88,155^. It should be noted that experiences from the five regions of Brazil show that CKD has been addressed in accordance with the SUS procedures, that is, through municipal and state protocols, technical notes, CIB deliberations, among others^156,157,158,159,160,161,162^. Accordingly, the World Health Organization recently included CKD in the list of priority chronic non-communicable conditions for financing, monitoring, education, prevention, and multisectoral and regional collaboration actions^163^.

With regard to secondary prevention actions, screening with serum creatinine and the urinary albumin-to-creatinine ratio is particularly noteworthy because it is highly cost-effective^13,14,15,16,164^, especially when associated with the timely use of nephroprotective medications available within the SUS, such as ACE inhibitors, angiotensin receptor blockers, and SGLT2 inhibitors^26,70^. Individuals who underwent at least one UACR test in the previous 12 months are more likely to be using nephroprotective drugs and to have better control of comorbidities such as hypertension, even after adjustment for multiple confounding variables^81^. However, low rates of UACR testing and underuse of ACE inhibitors, angiotensin receptor blockers, and SGLT2 inhibitors have been demonstrated in several services in Brazil^29,30^, reinforcing the importance of the recommendations contained in this consensus. According to the current guideline, the prescription of nephroprotective medication depends on knowledge of eGFR and albuminuria results^26^.

Another relevant challenge is the unequal distribution of nephrologists across the country, making it impossible to universally refer all cases classified as high or very high risk according to the CKD heat map^28,37^. Matrix support models involving nephrologists have demonstrated a positive impact on the quality of care, reducing unnecessary referrals and increasing adherence to CKD screening^86,87,88,89,90^. In fact, matrix support is widely recommended in SUS legislation and guidelines and is fundamental to increasing the problem-solving capacity of PHC, but there is still a lack of public policies to encourage the establishment of nephrologists in remote areas^24,25,26^.

Telehealth emerges as a strategic tool to expand access, reduce regional inequalities, and integrate healthcare teams, as already demonstrated in national and international studies^118,119,120^. Teleconsultations, tele-education, and telediagnosis can support both PHC and secondary care. Likewise, emerging technologies based on artificial intelligence, including clinical decision support algorithms and kidney risk prediction, are likely to become an important part of nephrology practice in the coming years^108,109,110,111,112,113^, especially as electronic medical records expand throughout the SUS. However, structural challenges persist, such as the incomplete computerization of primary health care units and the limited availability of broadband internet in rural areas and small municipalities.

It is recognized that the full implementation of the recommendations depends on the ability of the federal government and the states to provide technical and financial support to PHC^165,166^ and to integrate it more efficiently with secondary care level and hospital and KRT services. However, it is safe and recommended that PHC refer no more than 95% of individuals at risk or with CKD, reinforcing its central role in fighting CKD and other NCDs^25,36^. Indicator-driven management, as proposed in this document, represents a robust tool for monitoring results, identifying weaknesses, correcting courses, and promoting continuous improvement, even in contexts of budget restrictions^121,122,123,124,125,126,127,128,129,130^.

## CONCLUSION

The recommendations presented in this consensus constitute a strategic framework for the management of CKD in PHC within the SUS. They align scientific evidence, public policies, management tools, and regional experiences, providing managers with a practical and feasible guide to improve early diagnosis, expand access to nephroprotective therapies, strengthen care coordination, and reduce disease progression. The implementation of these actions requires an interfederative agreement, sustained investments, ongoing professional training, and the progressive incorporation of technologies and innovations.

## Data Availability

This manuscript was based on the review, synthesis, and interpretation of the scientific literature, legislation, and technical documents. No original datasets were generated.
