# Supplementary material for: Recommendations of the Brazilian Society of Nephrology for the management of chronic kidney disease in primary health care
Source: J Bras Nefrol. 2026 Oct 2;48(4):e20260093. doi: 10.1590/2175-8239-JBN-2026-0093en (PMC13638185; doi:10.1590/2175-8239-JBN-2026-0093en)
Supplement: Translation

# Translation: Recomendações da Sociedade Brasileira de Nefrologia para o manejo da doença renal crônica na atenção primária à saúde

**DOI:** 10.1590/2175-8239-JBN-2026-0093pt

**Authors:** Farid Samaan, Viviane Calice-Silva, Felipe Costa Neves, Pablo Rodrigues Costa Alves, Geraldo Bezerra da Silva, Lilian Pires de Freitas do Carmo, Verônica de Jesus Rodrigues Cardozo Costa, Máira de Faria Polcheira, René Scalet dos Santos, Patrícia Ferreira Abreu, Gianna Mastroianni Kirsztajn, José A. Moura-Neto

**Affiliations:** 1 Sociedade Brasileira de Nefrologia, São Paulo, SP, Brasil.; 2 Secretaria de Estado da Saúde de São Paulo, São Paulo, SP, Brasil.; 3 Instituto Dante Pazzanese de Cardiologia, São Paulo, SP, Brasil.; 4 Fundação Pró-Rim, Joinville, SC, Brasil.; 5 Escola Bahiana de Medicina e Saúde Pública, Salvador, BA, Brasil.; 6 Universidade Federal da Paraíba, João Pessoa, PB, Brasil.; 7 Universidade de Fortaleza, Fortaleza, CE, Brasil.; 8 Instituto Federal de Educação, Ciência e Tecnologia do Ceará, Fortaleza, CE, Brasil.; 9 Universidade Federal de Minas Gerais, Belo Horizonte, MG, Brasil.; 10 Hospital Evangélico de Belo Horizonte, Belo Horizonte, MG, Brasil.; 11 Secretaria Municipal de Saúde de Parauapebas, Parauapebas, PA, Brasil.; 12 Secretaria de Estado de Saúde do Distrito Federal, Brasília, DF, Brasil.; 13 Chronos Pesquisa Clínica, Taguatinga, DF, Brasil.; 14 Secretaria Municipal de Saúde de Curitiba, Curitiba, PR, Brasil.; 15 Universidade Federal de São Paulo, São Paulo, SP, Brasil.

**Keywords:** Atenção Primária à Saúde, Programas de Rastreamento, Educação Continuada, Assistência Ambulatorial, Doença Renal Crônica, Sistema Único de Saúde, Consenso

## Abstract

O Brasil é um país em franca transição epidemiológica e demográfica que possui grandes desafios para enfrentar as condições crônicas não transmissíveis. Dentre elas, destaca-se a doença renal crônica (DRC), que tem uma prevalência crescente e é responsável por 3–7% dos gastos em saúde de muitas nações. A DRC é considerada uma das doenças com maior potencial para a aplicação de medidas preventivas, por apresentar fatores de risco comuns, exames diagnósticos de baixo custo e ampla disponibilidade, história natural conhecida, tratamento factível e custo-efetividade comprovada das intervenções. Mesmo com muitas dificuldades, o Brasil é reconhecido internacionalmente por possuir um dos sistemas públicos de saúde mais robustos e inclusivos do mundo (Sistema Único de Saúde, SUS). Uma das razões para esse reconhecimento é a abrangência e o caráter multiprofissional da Atenção Primária à Saúde (APS) do SUS. Apesar de haver diretrizes brasileiras e internacionais sendo publicadas e atualizadas há mais de duas décadas, estudos recentes continuam evidenciando falhas na implementação da linha de cuidado à pessoa com DRC no Brasil. O objetivo deste documento foi elaborar recomendações práticas para que o gestor da APS possa incluir a DRC em sua rotina de trabalho, considerando as diretrizes vigentes, as leis brasileiras, os recursos financeiros e estruturais disponíveis e as características do SUS.

## INTRODUÇÃO

O Brasil é um país latino-americano de renda média alta, com dimensões continentais, que atravessa franca transição epidemiológica e demográfica^1,2^. Nas últimas décadas, o percentual de indivíduos com mais de 60 anos, em relação à população total, aumentou de 9% para 13%^3^. No mesmo período, as prevalências de adultos com obesidade, hipertensão arterial (HA) e diabetes mellitus (DM) passaram de 11% para 23%, de 21% para 25% e de 5% para 9%, respectivamente^4^.

Assim como muitas nações, o Brasil possui grandes desafios para enfrentar as doenças crônicas não transmissíveis (DCNT)^5,6^. Dentre elas, destaca-se a doença renal crônica (DRC), que consome 3–7% do orçamento da saúde de muitas nações^7–9^ e cujo aumento da frequência está intimamente relacionado ao aumento da expectativa de vida da população e ao crescimento das prevalências de obesidade, HA e DM^10–12^.

A DRC é considerada uma das doenças com maior potencial para a aplicação de medidas preventivas por apresentar, entre outras características, fatores de risco comuns, exames diagnósticos de baixo custo e ampla disponibilidade, história natural conhecida, tratamento factível uma vez diagnosticada e custo-efetividade comprovada das intervenções^13–16^.

O Brasil é um país com sistema de saúde duplo, no qual 100% da população tem direito a serviços públicos (incluindo diálise e transplante renal) e cerca de 25% das pessoas possuem algum plano privado de saúde^17–19^. Mesmo com muitas dificuldades, o sistema de saúde público brasileiro é reconhecido internacionalmente por ser um dos mais robustos e inclusivos do mundo (Sistema Único de Saúde, SUS)^20,21^. Uma das razões para esse reconhecimento é a abrangência e o caráter multiprofissional da Atenção Primária à Saúde (APS) do SUS^22,23^.

Apesar da existência de diretrizes brasileiras e internacionais de DRC, que vêm sendo publicadas e atualizadas há mais de duas décadas^24–28^, estudos recentes continuam evidenciando falhas importantes na implementação da linha de cuidado à pessoa com DRC no Brasil e no mundo^29–35^. O objetivo deste documento foi elaborar recomendações práticas para que o gestor da APS possa incluir a DRC em sua rotina de trabalho, considerando as diretrizes vigentes, os recursos disponíveis e as características do SUS brasileiro.

## MÉTODOS

O presente documento é um posicionamento institucional da Sociedade Brasileira de Nefrologia (SBN), de caráter educativo e não normativo.

### Identificação de processos-chave

A primeira fase da elaboração deste documento foi a identificação de ações essenciais que o gestor da APS deve realizar com o objetivo de incluir a DRC em sua rotina de trabalho. Para essa etapa, foram consultados membros da Diretoria Executiva, do Departamento de Epidemiologia e Prevenção de DRC e médicos associados da SBN, com experiência em gestão pública e/ou matriciamento de equipes de APS, sendo pelo menos um representante de cada região do Brasil.

### Elaboração de recomendações

Sempre que possível, as recomendações foram elaboradas com base em evidências científicas e legislações brasileiras. Algumas recomendações eram de caráter técnico, permitindo que fossem amparadas por literatura especializada e normas vigentes. Outras, entretanto, por serem de caráter essencialmente gerencial, demandaram a elaboração de sugestões com base na experiência dos autores.

### Consulta pública

A primeira versão das recomendações foi disponibilizada para consulta pública, divulgada nas páginas eletrônicas e redes sociais da Sociedade Brasileira de Nefrologia. Os respondentes foram convidados a acessar um formulário eletrônico, no qual assinalaram seu posicionamento de maneira categorizada (“concordo”, “concordo parcialmente” ou “discordo”) e tiveram espaço para elaborar suas contribuições em campo livre, sem restrição quanto ao número de sugestões por participante.

## RESULTADOS

Os processos-chave identificados para que a DRC seja incluída na rotina de trabalho da APS foram: (1) responsabilidade da União; (2) responsabilidades de estados e municípios; (3) aprovação de um protocolo municipal de rastreamento da DRC na APS; (4) padronização dos laudos dos exames diagnósticos da DRC; (5) treinamento profissional; (6) coordenação do cuidado integral em todos os estágios de DRC; e (7) gestão por indicadores (Quadro 1).

**Quadro 1.** Processos-chave para manejo da DRC na APS.

Processo-chave	Recomendações
Responsabilidades da União	– Formular a política pública para enfrentamento da DRC na APS. – Apoiar financeiramente estados e municípios na execução de ações e serviços de saúde. – Manter atualizada a Tabela de Procedimentos e Medicamentos do SUS.
Responsabilidades dos estados	– Prover apoio técnico e financeiro para implementação das políticas nacionais de DRC nos municípios. – Monitorar as ações da APS em DRC. – Garantir o acesso à atenção especializada em Nefrologia nos níveis secundário e terciário.
Responsabilidades dos municípios	– Executar as ações e serviços de saúde na APS. – Priorizar a ESF na organização da APS. – Garantir espaços de participação multiprofissional e da população. – Incluir a DRC na rotina de trabalho: aprovar um protocolo municipal, realizar treinamentos profissionais recorrentes, utilizar indicadores e metas.
Aprovação de Protocolo de DRC na APS	O protocolo de DRC na APS deve incluir, no mínimo, o seguinte conteúdo: – A população-alvo para o rastreamento. – Os exames de rastreamento. – Os critérios de encaminhamento para atenção especializada em nefrologia.
Padronização de laudos de exames	– O resultado de CrS (código SUS: 02.02.01.031-7) deve ser acompanhado da TFGe. – O resultado de microalbuminúria (código SUS: 02.02.05.009-2) deve ser acompanhado da RAC.
Treinamento profissional	O treinamento profissional deve seguir os seguintes princípios: – Legitimidade: considerar a PNAB, o PMS e a PAS vigentes e ser baseado no protocolo municipal. – Abrangência multiprofissional: médicos, enfermeiros, nutricionistas, dentistas, farmacêuticos, assistentes sociais, agentes comunitários de saúde, entre outros. – Viabilidade e recorrência: os treinamentos devem ter duração de 120 minutos e ser realizados a cada seis meses (sugestão). – Diálogo: os treinamentos devem permitir participação ativa dos profissionais da assistência direta.
Coordenação do cuidado integral na APS	As pessoas encaminhadas para a atenção secundária e/ou terciária devem manter o vínculo com a APS.
Gestão por indicadores	O gestor público municipal deve monitorar um ou mais dos seguintes indicadores de qualidade da assistência à DRC na APS: – % de adultos com HA e/ou DM e ≥ 1 dosagem de CrS em 12 meses. – % de adultos com HA e/ou DM e ≥ 1 dosagem de RAC em 12 meses. – % de adultos com HA e pressão arterial < 130 x 80 mmHg. – % de adultos com DM e HbA1c < 7,0%. – % de adultos com DRC em uso de IECA ou BRA, exceto se contraindicado. – % de adultos com DRC em uso de inibidores de SGLT2, exceto se contraindicado.

Abreviaturas – DRC: doença renal crônica; APS: atenção primária à saúde; SUS: Sistema Único de Saúde; CrS: creatinina sérica; ESF: Estratégia de Saúde da Família; TFGe: taxa de filtração glomerular estimada; RAC: relação albumina/creatinina em amostra isolada de urina; PNAB: Política Nacional de Atenção Básica; PMS: Plano Municipal de Saúde; PAS: Programação Anual de Saúde; HA: hipertensão arterial; DM: diabetes mellitus; HbA1c: hemoglobina glicada; IECA: inibidores da enzima conversora de angiotensina; BRA: bloqueadores do receptor de angiotensina; SGLT2: cotransportador sódio-glicose tipo 2.

### Responsabilidades da união

**Recomendação 1**. O Ministério da Saúde (MS) deve determinar as diretrizes nacionais para o manejo da DRC na APS e apoiar financeiramente estados e municípios na execução de procedimentos diagnósticos e terapêuticos, mantendo atualizada a tabela de valores repassados aos prestadores de serviço. Por ser uma condição comum, prevenível e onerosa, a DRC deve ser incluída em todas as propostas inovadoras do MS, tais como aquelas relacionadas ao prontuário eletrônico do SUS e à remuneração por desempenho (indicadores de prevenção em saúde, oferta de cuidados integrados, entre outras).

**Comentário**. O ente federal é o responsável por elaborar as diretrizes que regem as políticas públicas de saúde. Entre os documentos mais recentes relacionados à DRC no Brasil estão a Portaria n° 1675, de 2018^25^, que contém os critérios para organização do cuidado à pessoa com DRC no SUS, e o Protocolo Clínico e Diretrizes Terapêuticas (PCDT) de DRC de 2024^26^, o qual descreve as estratégias para atenuar a progressão da DRC. Em ambas as legislações brasileiras, os papéis da APS no diagnóstico precoce, no tratamento das fases iniciais da DRC e na coordenação do cuidado integral ao paciente estão destacados. O PCDT de DRC, entre outras recomendações, apresenta normativas técnicas específicas para as ações de rastreamento da DRC e para o uso de medicações no âmbito do SUS.

### Responsabilidades dos estados e municípios

**Recomendação 2**. Todo município do Brasil deve ter uma rotina definida para o enfrentamento da DRC em nível primário de atenção à saúde, independentemente do número de habitantes. Essa rotina inclui, no mínimo, duas ações: a elaboração de um protocolo de rastreamento na população de risco e o manejo de casos de DRC de baixo e moderado risco. As ações de saúde em DRC nos níveis secundário e terciário devem ser pactuadas regionalmente de maneira bipartite (estados e municípios) e incluem matriciamento, assistência especializada em nefrologia ambulatorial e em terapia renal substitutiva (TRS).

**Comentário**. De acordo com a Política Nacional de Atenção Básica (PNAB), a APS do SUS está essencialmente sob gestão dos municípios, os quais têm autonomia para planejar e executar as ações e serviços de saúde^36^. O PCDT de DRC de 2024 determina que indivíduos de risco para a DRC sejam rastreados para a doença em nível primário^26^. No Brasil, cerca de 30% dos adultos possuem um ou mais fatores de risco para DRC (HA, DM, obesidade, doença cardiovascular estabelecida, entre outros), o que justifica o fato de a APS ser absolutamente essencial para o diagnóstico precoce da doença^4^. Quando um município ou região realiza os exames de rastreamento de DRC recomendados, estima-se que mais de 97% das pessoas não apresentem a condição ou possuam DRC de baixo e moderado risco, de acordo com o mapa de calor da doença^32,37,38^ (Tabela 1). Desse modo, os municípios precisam estar preparados para assistir à grande maioria dos pacientes rastreados.

**Tabela 1.** Distribuição percentual das categorias de risco de DRC em adultos.

Estágio DRC	TFGe (mL/min/1,73 m²)	Nível de RAC (mg/g)			Total
		Leve < 30	Moderada 30-300	Grave > 300	
1	≥ 90	45,01 (sem DRC)	1,99	0,37	47,37
2	60–89	40,72 (sem DRC)	1,78	0,31	42,81
3 a	45–59	7,41	0,55	0,13	8,09
3 b	30–44	1,19	0,21	0,07	1,47
4	15-29	0,10	0,05	0,05	0,20
5	< 15	0,03	0,01	0,02	0,06
Total		94,46	4,59	0,95	100,00

Abreviaturas – DRC: doença renal crônica; TFGe: taxa de filtração glomerular estimada; RAC: relação albumina/creatinina em amostra isolada de urina.

Notas – Cada célula colorida da figura contém a distribuição percentual (%) da categoria de risco de DRC correspondente (Ref. 37). Categorias de risco de DRC, segundo o KDIGO (*Kidney Disease: Improving Global Outcomes*) (Ref. 28): verde, risco baixo; amarelo, risco moderado; laranja, risco alto; vermelho, risco muito alto.

No Brasil, 69% dos municípios têm menos de 20.000 habitantes, percentual que varia entre 57% e 77% nas cinco regiões do país (Tabela 2)^39,40^. Portanto, a atenção especializada em nefrologia nos níveis secundário e terciário deve ser garantida e organizada, principalmente, pelos estados e pelo governo federal. A Portaria de parâmetros assistenciais do Ministério da Saúde vigente recomenda a oferta de 1.600 consultas com nefrologista por ano para cada 100.000 habitantes^39^.

**Tabela 2.** Distribuição dos municípios brasileiros segundo estimativa populacional em 2025 [40].

Região/UF	Frequência de municípios de acordo com número de habitantes, % (n)					
	Menos de 20.000	20.000 a 49.999	50.000 a 99.999	100.000 a 499.999	500.000 ou mais	Total
Norte	57,3 (258)	26,7 (120)	9,6 (43)	5,6 (25)	0,9 (4)	100,0 (450)
AC	63,6 (14)	27,3 (6)	4,5 (1)	4,5 (1)	0,0 (0)	100,0 (22)
AM	35,5 (22)	45,2 (28)	12,9 (8)	4,8 (3)	1,6 (1)	100,0 (62)
AP	68,8 (11)	18,8 (3)	0,0 (0)	12,5 (2)	0,0 (0)	100,0 (16)
PA	27,1 (39)	43,1 (62)	19,4 (28)	9,0 (13)	1,4 (2)	100,0 (144)
RO	65,4 (34)	21,2 (11)	5,8 (3)	5,8 (3)	1,9 (1)	100,0 (52)
RR	53,3 (8)	40,0 (6)	0,0 (0)	6,7 (1)	0,0 (0)	100,0 (15)
TO	93,5 (130)	2,9 (4)	2,2 (3)	1,4 (2)	0,0 (0)	100,0 (139)
Nordeste	66,3 (1190)	23,4 (419)	6,5 (116)	3,2 (58)	0,6 (11)	100,0 (1794)
AL	61,7 (63)	28,4 (29)	7,8 (8)	1,0 (1)	1,0 (1)	100,0 (102)
BA	60,4 (252)	28,5 (119)	6,7 (28)	3,8 (16)	0,5 (2)	100,0 (417)
CE	46,7 (86)	32,6 (60)	15,8 (29)	4,3 (8)	0,5 (1)	100,0 (184)
MA	59,0 (128)	30,9 (67)	5,5 (12)	4,1 (9)	0,5 (1)	100,0 (217)
PB	85,2 (190)	9,9 (22)	3,1 (7)	1,3 (3)	0,4 (1)	100,0 (223)
PE	45,9 (85)	35,1 (65)	10,8 (20)	7,0 (13)	1,1 (2)	100,0 (185)
PI	87,9 (197)	9,4 (21)	1,8 (4)	0,4 (1)	0,4 (1)	100,0 (224)
RN	83,2 (139)	10,8 (18)	3,6 (6)	1,8 (3)	0,6 (1)	100,0 (167)
SE	66,7 (50)	24,0 (18)	2,7 (2)	5,3 (4)	1,3 (1)	100,0 (75)
Centro-Oeste	72,2 (338)	17,7 (83)	4,3 (20)	4,7 (22)	1,1 (5)	100,0 (468)
DF	0,0 (0)	0,0 (0)	0,0 (0)	0,0 (0)	100,0 (1)	100,0 (1)
GO	76,4 (188)	13,4 (33)	3,3 (8)	6,1 (15)	0,8 (2)	100,0 (246)
MS	55,7 (44)	35,4 (28)	5,1 (4)	2,5 (2)	1,3 (1)	100,0 (79)
MT	74,6 (106)	15,5 (22)	5,6 (8)	3,5 (5)	0,7 (1)	100,0 (142)
Sudeste	67,1 (1120)	17,3 (289)	6,2 (103)	8,0 (133)	1,4 (23)	100,0 (1668)
ES	51,3 (40)	33,3 (26)	2,6 (2)	10,3 (8)	2,6 (2)	100,0 (78)
MG	77,7 (663)	13,8 (118)	4,2 (36)	3,8 (32)	0,5 (4)	100,0 (853)
RJ	28,3 (26)	31,5 (29)	8,7 (8)	23,9 (22)	7,6 (7)	100,0 (92)
SP	60,6 (391)	18,0 (116)	8,8 (57)	11,0 (71)	1,6 (10)	100,0 (645)
Sul	76,7 (913)	13,4 (159)	5,2 (62)	4,4 (52)	0,4 (5)	100,0 (1191)
PR	75,7 (302)	14,8 (59)	3,5 (14)	5,5 (22)	0,5 (2)	100,0 (399)
RS	78,5 (390)	12,3 (61)	5,4 (27)	3,6 (18)	0,2 (1)	100,0 (497)
SC	74,9 (221)	13,2 (39)	7,1 (21)	4,1 (12)	0,7 (2)	100,0 (295)
Total	68,6 (3819)	19,2 (1070)	6,2 (344)	5,2 (290)	0,9 (48)	100,0 (5571)

Abreviaturas – UF, unidade da federação. Região Norte: AC, Acre; AM, Amazonas; AP, Amapá; RO, Rondônia; RR, Roraima; TO, Tocantins. Região Nordeste: AL, Alagoas; BA, Bahia; CE, Ceará; MA, Maranhão; PB, Paraíba; PE, Pernambuco; PI, Piauí; RN, Rio Grande do Norte; SE, Sergipe. Região Centro-Oeste: GO, Goiás; DF, Distrito Federal; MS, Mato Grosso do Sul; MT, Mato Grosso. Região Sudeste: ES, Espírito Santo; MG, Minas Gerais; RJ, Rio de Janeiro; SP, São Paulo. Região Sul: PR, Paraná; RS, Rio Grande do Sul; SC, Santa Catarina.

Nota – Cada célula da tabela contém as frequências relativa e absoluta de municípios de acordo com o número de habitantes apontado nas colunas.

Reconhece-se a seguinte tendência do gasto relativo em saúde pelos três entes federativos no Brasil (município, estado e União): aumento da participação dos municípios, redução do aporte da União e estabilidade do gasto dos estados^41,42^. Muitos municípios brasileiros podem apresentar dificuldade em financiar procedimentos diagnósticos simples, como os da rotina destinados a pessoas com HA e DM, sendo obrigatório, assim, o aporte financeiro do estado e/ou da União para tal fim. Entretanto, mesmo nesses casos, a execução de ações em nível primário de atenção à saúde continua sendo de responsabilidade do ente municipal^36^.

### Protocolo municipal de rastreamento da DRC na APS

Embora existam diretrizes brasileiras e internacionais para o manejo da DRC^24,26–28^, sabe-se que a elaboração de rotinas internas por parte dos municípios representa o resultado da divulgação e discussão do tema entre gestores e profissionais da assistência. A elaboração de um protocolo municipal permite que sejam realizadas adaptações que respeitem as diversidades de natureza econômica, cultural, social e de acesso aos serviços de saúde existentes no Brasil.

O protocolo municipal de rastreamento da DRC na APS deve nortear as atividades dos profissionais assistentes, informando, de forma clara, no mínimo, as respostas às seguintes questões: Qual é a população-alvo? Quem pode solicitar os exames? Quais exames devem ser solicitados e com qual periodicidade? Quando encaminhar casos de DRC para o nefrologista?

**Recomendação 3**. A população-alvo para rastreamento da DRC na APS deve ser constituída por adultos com um ou mais dos seguintes fatores de risco: HA, DM, obesidade, tabagismo, idade avançada (≥ 60 anos), doença cardiovascular ou cerebrovascular estabelecida, história de DRC em familiares de primeiro grau, uso crônico de medicações nefrotóxicas, doenças autoimunes com acometimento sistêmico e patologias crônicas do trato urinário (infecção ou cálculos de repetição, hiperplasia ou neoplasia prostática, disfunção vesical crônica, entre outras).

**Comentário**. As principais justificativas para que indivíduos, mesmo assintomáticos, com um ou mais fatores de risco realizem exames de triagem de DRC são: a prevalência de DRC em adultos é de 8–14% no mundo^43^; estima-se que apenas 10–20% das pessoas com DRC saibam que têm esse diagnóstico^44^; a DRC pode não apresentar sintomas em suas fases iniciais, o que acontece, sobretudo, devido à capacidade de adaptação dos rins à perda de néfrons^45^; a presença e a gravidade da DRC estão fortemente relacionadas com eventos cardiovasculares, internações, necessidade de TRS, óbito, prejuízo da qualidade de vida e gastos em saúde^46–48^. É preciso destacar que, de acordo com diretrizes brasileiras e consensos internacionais, o rastreamento de DRC está indicado somente para indivíduos com fatores de risco para a doença^24–28^. Até o momento, não se recomenda a realização de exames de avaliação renal para pessoas sem fatores de risco para DRC e com idade inferior a 60 anos^49^. Embora não haja evidência científica para o rastreamento de DRC nessa população, a realização anual de testes de creatinina sérica e elementos anormais do sedimento urinário (EAS) na população geral pode ser considerada de baixo custo em alguns contextos e pode ser útil para a identificação de casos de DRC de origem desconhecida.

**Recomendação 4**. Os médicos que atuam na APS devem ser capacitados para solicitar os exames de rastreamento da DRC, de acordo com os protocolos assistenciais vigentes (TFGe e RAC).

**Comentário**. O detalhamento dos papéis e responsabilidades das diferentes categorias profissionais da APS deve ser definido em nível municipal, em consonância com a PNAB e com a legislação vigente^36^. A organização das ações de rastreamento e acompanhamento da DRC deve respeitar os fluxos assistenciais locais, a capacidade instalada dos serviços e a coordenação do cuidado no âmbito da APS. Experiências descritas em diferentes regiões do país demonstram que a estruturação de ações protocolares para avaliação de condições prevalentes e para o rastreamento de doenças crônicas, integradas a fluxos assistenciais bem definidos e com retaguarda médica, contribui para ampliar o acesso e a resolubilidade da APS^50–54^. O rastreamento da DRC se justifica pela elevada prevalência de fatores de risco facilmente identificáveis na APS, como hipertensão arterial e diabetes mellitus, e pela disponibilidade de exames de baixo custo, amplamente acessíveis no SUS e não invasivos^18^.

**Recomendação 5**. Todos os municípios devem padronizar, em seu protocolo de DRC na APS, a realização de dois exames para rastreamento da doença: creatinina sérica (código SUS: 02.02.01.031-7) e relação albumina/creatinina em amostra isolada de urina (RAC) (códigos SUS: dosagem de “microalbumina” na urina, 02.02.05.009-2, e creatinina urinária, 02.02.01.031-7).

**Recomendação 6**. Os gestores públicos, em seus serviços próprios ou de terceiros, devem pactuar com os laboratórios que: (1) todo resultado de creatinina sérica (CrS), realizado para indivíduos com mais de 18 anos, deve ser acompanhado da taxa de filtração glomerular estimada pela equação CKD-EPI (*Chronic Kidney Disease Epidemiology Collaboration*) de 2021 (TFGe, em mL/min/1,73 m^2^); e (2) todo resultado de “microalbuminúria” deve ser acompanhado da relação albumina/creatinina (RAC, em mg/g).

**Comentário**. Creatinina sérica (código SUS: 02.02.01.031-7) e dosagem de “microalbumina” na urina ou microalbuminúria (código SUS: 02.02.05.009-2) são nomenclaturas contidas na lista de procedimentos diagnósticos e terapêuticos oferecidos no SUS, isto é, estão padronizadas no Sistema de Gerenciamento da Tabela de Procedimentos, Medicamentos e OPM (Órteses, Próteses e Materiais) (SIGTAP)^18^. Embora a creatinina sérica seja o marcador de função renal mais conhecido e disponível no Brasil e no mundo, esse biomarcador sofre influência, entre outros fatores, da massa muscular do indivíduo^55,56^. Por isso, há mais de quatro décadas, a comunidade científica tem desenvolvido equações para calcular a TFGe a partir da creatinina sérica, utilizando variáveis como idade, sexo, raça, peso, entre outras^57–60^. Estudos demonstraram que incluir a informação automática de TFGe nos laudos de creatinina sérica pode aumentar a identificação das fases iniciais da doença, elevar o uso de medicamentos nefroprotetores e reduzir a prescrição de drogas nefrotóxicas^61^. A fórmula atualmente recomendada para estimar a TFG de adultos a partir da creatinina sérica é a CKD-EPI de 2021, a qual requer apenas as variáveis idade e sexo (Figura 1)^26,28,60^.

**Figura 1.** Modelo de laudo do exame de creatinina sérica (código SUS: 02.02.01.031-7) para indivíduos com mais de 18 anos.

A TFGe não é suficiente para o diagnóstico precoce e a estratificação de risco da DRC^62–64^. Todas as políticas públicas de enfrentamento da DRC devem garantir recursos físicos e treinamento profissional para que um marcador urinário de prognóstico da doença seja adicionado à rotina de seu rastreamento, em complemento à determinação da TFGe^26,28^. O melhor marcador de DRC na prática clínica é a proteinúria, e a proteína urinária mais importante para a rotina da APS é a albumina^65,66^. Recomenda-se que a albuminúria, assim como ocorre com outros biomarcadores urinários, seja normatizada pela creatinina urinária^67^. Desse modo, o termo “microalbuminúria” não é adequado, e recomenda-se que seja substituído por RAC na tabela de procedimentos do SUS^28,65,66^. Para a dosagem de RAC, os laboratórios que prestam serviço ao SUS podem efetuar a cobrança de dois códigos da Tabela SIGTAP: 02.02.05.009-2 (microalbuminúria) e 02.02.01.031-7 (dosagem de creatinina urinária) (Figura 2). As dosagens sérica e urinária de creatinina possuem o mesmo código na Tabela SIGTAP (02.02.01.031-7).

**Figura 2.** Modelo de laudo do exame de “microalbuminúria” (códigos SUS: 02.02.05.009-2 e 02.02.01.031-7).

Existem diversos métodos de avaliação de proteinúria e, embora haja boa equivalência entre eles, para finalidade de triagem de DRC, a dosagem em amostra isolada de urina é preferível às análises em 24 horas^27–29^. A RAC tem maior custo que os exames EAS e RPC, mas seu custo relativo é baixo frente ao montante do que é gasto rotineiramente com exames de análises clínicas para pessoas com HA ou DM no SUS^18,68^. Ademais, e principalmente, a RAC é mais sensível para o diagnóstico precoce e prognóstico de DRC que os demais métodos em amostra isolada^69^ (Tabela 3).

**Tabela 3.** Equivalência dos níveis de proteinúria [27,65].

Nome do exame	Tipo de dosagem de proteínas	Nível de proteinúria		
		Leve ou ausente	Moderada	Grave
EAS, sumário de urina ou urina tipo 1 (fita reagente)	Qualitativa	Ausente/traços	1+	2+ ou 3+
RAC (mg/g)	Quantitativa	<30	30–300	>300
RPC (g/g)	Quantitativa	<0,2	0,2–1,0	>1,0
Albuminúria 24 horas (mg/24h)	Quantitativa	<30	30–300	>300
Proteinúria 24 horas (mg/24h)	Quantitativa	<150	150–500	>500

Abreviaturas – EAS: elementos anormais do sedimento urinário; RAC: relação albumina/creatinina; RPC: relação proteína/creatinina.

A relevância da TFGe e da RAC para a rotina da assistência à DRC no SUS é reforçada pela necessidade de se conhecer os resultados desses exames não só para diagnóstico e estratificação de risco, mas também para a prescrição e a solicitação de medicamentos nefroprotetores essenciais (inibidores da enzima conversora de angiotensina, bloqueadores do receptor de angiotensina e antagonistas dos receptores de mineralocorticoide) e do componente especializado (inibidores do cotransportador sódio-glicose tipo 2)^26,70^ (Quadro 2).

**Quadro 2.** Medicamentos nefroprotetores disponíveis no sistema único de saúde (SUS) em 2025 [26,70].

Classe	Medicamento	Indicações para DRC	Contraindicações
IECA	– Captopril – Enalapril	– DM: RAC > 30 mg/g – Não DM: RAC ≥ 300 mg/g	– Hiperpotassemia refratária – Estenose de artéria renal bilateral
BRA	– Losartana – Valsartana	– DM: RAC > 30 mg/g – Não DM: RAC ≥ 300 mg/g	– Hiperpotassemia refratária – Estenose de artéria renal bilateral
Inibidores de SGLT2	Dapagliflozina	– DM: TFGe 25-75 mL/min/1,73 m² – Não DM: TFGe 25-75 mL/min/1,73 m² e RAC ≥ 300 mg/g	– DM tipo 1 – Paciente em diálise
Antagonista dos receptores de mineralocorticoide	Espironolactona	– TFGe > 15 mL/min/1,73 m² e RAC ≥ 300 mg/g	Hiperpotassemia refratária

Abreviaturas – IECA: inibidores da enzima conversora de angiotensina; BRA: bloqueadores do receptor de angiotensina; SGLT2: cotransportador sódio-glicose tipo 2; DM: diabetes mellitus; RAC: relação albumina/creatinina urinária; TFGe: taxa de filtração glomerular estimada.

**Recomendação 7**. O município em que residam pessoas sem acesso à rede laboratorial tradicional deve considerar a utilização de exames de rastreamento de DRC realizados no local de atendimento.

**Comentário**. A utilização de exames no local de atendimento (*point-of-care*) é endossada pelo consenso internacional de DRC vigente (*Kidney Disease: Improving Global Outcomes* [KDIGO] 2024) e por diretrizes locais^28,71^. É sabido que populações vulneráveis e com acesso limitado a serviços de saúde básicos, como coleta de exames de análises clínicas, apresentam maior risco e gravidade de DRC^72–75^. Deve-se destacar que o benefício e a viabilidade de dosagens de creatinina sérica e proteinúria no local de atendimento foram demonstrados em diferentes países, independentemente do nível de renda da nação^76–79^. No Brasil, essa estratégia mostrou-se igualmente factível. Em março de 2025, durante a campanha do Dia Mundial do Rim, a Sociedade Brasileira de Nefrologia realizou ações de rastreamento com testes rápidos em 40 cidades de 19 estados e no Distrito Federal, envolvendo 8.374 pessoas avaliadas. Dentre essas, 3.370 indivíduos (40,2%) apresentaram alteração da creatinina, sendo encaminhados para confirmação diagnóstica e seguimento clínico. Esses resultados reforçam a segurança, viabilidade e aplicabilidade da estratégia no contexto brasileiro^80^.

**Recomendação 8**. O número mínimo recomendado de consultas médicas por ano para pacientes com DRC deve variar de acordo com a estratificação de risco da doença, realizada pelo mapa de risco ou mapa de calor: risco baixo (1 vez por ano), risco moderado (2 vezes por ano), risco alto (3 vezes por ano), risco muito alto (4 vezes por ano; 6–12 vezes por ano se DRC estágio 5).

**Comentário**. Estudos demostraram que o mapa de risco ou mapa de calor da DRC apresenta associação significativa com eventos renais e não renais, entre eles: progressão da DRC, necessidade de TRS, eventos cardiovasculares (infarto agudo do miocárdio, acidente vascular cerebral, arritmias, doença arterial periférica), hospitalização e óbito por todas as causas^81^. Desse modo, justifica-se o aumento progressivo da frequência de acompanhamento médico à medida que a categoria de risco da DRC se eleva. A frequência de consultas deve também ser ajustada em função da presença de sintomas e da velocidade de progressão da DRC.

**Recomendação 9**. Os municípios devem adotar a rotina de encaminhar para avaliação com nefrologista os casos de DRC que apresentem as categorias de risco alto e muito alto do mapa de calor.

**Comentário**. Estima-se que cerca de 3% dos indivíduos rastreados com TFGe e RAC apresentem categorias de risco alto ou muito alto no mapa de calor da DRC (Tabela 1)^28,37^. Esse contingente de pessoas é elevado frente à disponibilidade de médicos nefrologistas em algumas regiões do país^82^. Por essa razão, diversas diretrizes e protocolos adotam como critério prioritário de encaminhamento para a atenção especializada os indivíduos com DRC nos estágios 4 e 5, os quais correspondem a aproximadamente 0,3% da população rastreada^26,37^. Essa estratégia busca compatibilizar a necessidade de cuidado especializado com a capacidade instalada dos sistemas de saúde.

Entretanto, o presente consenso da Sociedade Brasileira de Nefrologia propõe uma abordagem mais abrangente e, sempre que possível, um encaminhamento mais precoce, incluindo também os indivíduos em estágio 3b e aqueles com albuminúria grave (A3), mesmo na presença de TFGe preservada. Essa recomendação baseia-se em evidências consistentes de que a albuminúria e a redução mais acentuada da TFGe são marcadores independentes de pior prognóstico renal e cardiovascular, estando associadas a maior risco de necessidade de TRS, eventos cardiovasculares, internações e óbito^83–85^. Adicionalmente, sabe-se que a conscientização dos profissionais de saúde sobre a importância do rastreamento da DRC com avaliação de RAC ainda é limitada no Brasil e em outros países^29,86^. Como consequência, muitos casos de pacientes com RAC ≥ 30 mg/g e TFGe preservada permanecem não reconhecidos, retardando o diagnóstico e o tratamento oportuno de nefropatias hipertensiva e diabética, bem como de glomerulopatias primárias.

**Recomendação 10**. O acompanhamento de pacientes com DRC na APS deve incluir, mas não se restringir a: (1) orientações para modificação do estilo de vida; (2) prescrição de medicações que retardam a progressão da DRC, quando indicado; (3) avaliação e tratamento de comorbidades e complicações da DRC; e (4) não usar anti-inflamatórios não esteroidais.

**Comentário**. Considerando a Recomendação 9, cerca de 97% dos pacientes rastreados para DRC deverão ser acompanhados somente na APS (Tabela 1); caso o município opte por uma rotina mais restritiva, isto é, recomendar a referência para nefrologista somente dos casos de DRC nos estágios 4 e 5, esse percentual ultrapassará 99%. Em ambos os casos, devem existir rotinas claras para o manejo da DRC na APS.

Os exames recomendados para manejo da DRC na APS devem incluir, mas não se restringir a: hemograma, creatinina sérica, EAS, RAC, glicemia de jejum, hemoglobina glicada (em DM), colesterol total e frações, triglicérides, potássio, dosagem de 25-hidroxi-vitamina D, cálcio, fósforo, perfil de ferro (ferro, ferritina e transferrina), paratormônio e sorologias para hepatites B e C, e HIV. A sugestão de intervalo de tempo para realização desses exames está descrita na Tabela 4, entretanto poderá variar de acordo com o caso clínico específico do paciente^24,26–28^. Deve-se considerar a inclusão de rotina da dosagem de sódio sérico (diante do elevado percentual de indivíduos em uso de diuréticos) e do VDRL (considerando a elevada prevalência de sífilis na população brasileira e sua associação com glomerulopatias).

**Tabela 4.** Exames recomendados para acompanhamento da DRC (adaptado das refs. 24, 26-28).

Estágio DRC	Procedimentos	Intervalo	Nível de atenção
1 e 2 (TFGe ≥ 60)	– TFGe, EAS, RAC e K – Hemograma – Glicemia, HbA1c (se DM) – Colesterol total e frações, triglicérides	12 meses	Seguir na APS Encaminhar para AEA se RAC ≥ 300
3 a (TFGe 45-59)	– TFGe, EAS, RAC e K – Hemograma – Glicemia, HbA1c (se DM) – Ferro, ferritina, transferrina – Colesterol total e frações, triglicérides – HBsAg, Anti-HBs, Anti-HBc-total – Anti-HCV, Anti-HIV	12 meses	Seguir na APS Encaminhar para AEA se RAC ≥ 30
3 b (TFGe 30-44)	– TFGe, EAS, RAC e K – Hemograma – Glicemia, HbA1c (se DM) – Ferro, ferritina, transferrina	6 meses	Encaminhar para AEA Manter seguimento na APS
	– Colesterol total e frações, triglicérides – Ca, P, PTH, 25-OH-vitamina D – HBsAg, Anti-HBs, Anti-HBc-total – Anti-HCV, Anti-HIV	12 meses	
4 (TFGe 15-29)	– TFGe, EAS, RAC e K – Hemograma – Glicemia, HbA1c (se DM) – Ferro, ferritina, transferrina	3 meses	Encaminhar para AEA Manter seguimento na APS
	– Colesterol total e frações, triglicérides – Ca, P, PTH, 25-OH-vitamina D – Proteínas totais e frações	6 meses	
	– ECG, Radiografia tórax – Ultrassom de rins e vias urinárias – HBsAg, Anti-HBs, Anti-HBc-total – Anti-HCV, Anti-HIV	12 meses	
5 (TFGe < 15)	– Hemoglobina, hematócrito – Ureia, creatinina, Na, K, Ca, P – Gasometria venosa	1 mês	Encaminhar para AEA Manter seguimento na APS
	– Hemograma – Ferro, ferritina, transferrina	3 meses	
	– Glicemia, HbA1c (se DM) – Colesterol total e frações, triglicérides – Ca, P, PTH, 25-OH-vitamina D – Proteínas totais e frações	6 meses	Encaminhar para AEA Manter seguimento na APS
	– ECG, Radiografia tórax – Ultrassom de rins e vias urinárias – HBsAg, Anti-HBs, Anti-HBc-total – Anti-HCV, Anti-HIV	12 meses	

Abreviaturas – DRC: doença renal crônica; TFGe: taxa de filtração glomerular estimada (em mL/min/1,73 m²); EAS: elementos anormais do sedimento urinário (sinônimos: sumário de urina, urina tipo 1); RAC: relação albumina/creatinina urinária (em mg/g); K: potássio; HbA1c: hemoglobina glicada; DM: diabetes mellitus; Ca: cálcio; P: fósforo; PTH: paratormônio; Na: sódio; ECG: eletrocardiograma; APS: atenção primária à saúde; AEA: atenção especializada ambulatorial em Nefrologia; HBsAg: antígeno de superfície do vírus da hepatite B; Anti-HBs, anticorpo contra o antígeno de superfície do vírus da hepatite B; Anti-HBc-total: anticorpo contra o antígeno nuclear do vírus da hepatite B; Anti-HCV: anticorpo contra o vírus da hepatite C; Anti-HIV: anticorpo contra o vírus HIV; HIV: vírus da imunodeficiência humana.

### Treinamento profissional

**Recomendação 11**. O tema da DRC deve fazer parte de programas de Educação Permanente em Saúde (EPS) do SUS. O conteúdo e o formato dos treinamentos profissionais devem ser definidos conjuntamente por profissionais da APS e da Atenção Especializada Ambulatorial (AEA) em Nefrologia.

**Comentário.** A EPS é uma estratégia do SUS regulamentada pelo Ministério da Saúde e definida na Política Nacional de Educação Permanente em Saúde (PNEPS)^87^. Considerando a ausência de nefrologistas na maioria dos municípios brasileiros, os estados devem garantir apoio técnico e educacional aos municípios para que a resolubilidade das ações na APS seja maximizada. A AEA é responsável pelo matriciamento da APS^25,26^. As atividades de EPS, no contexto da DRC, devem ser pactuadas nos grupos técnicos bipartites (CIB, Comissão Intergestores Bipartite) e regionais (CIR, Comissão Intergestores Regional)^88^.

A justificativa para que os treinamentos em DRC sejam elaborados conjuntamente entre nefrologistas e profissionais da APS relaciona-se ao fato de que esses últimos são os que, em geral, possuem maior experiência com a assistência integral aos pacientes, maior vivência do trabalho em equipe e maior conhecimento das condições de vida dos pacientes e dos determinantes sociais das doenças no território^89,90^.

**Recomendação 12**. Sempre que possível, o treinamento profissional em DRC deve ser realizado com múltiplas categorias profissionais da APS, o que inclui, mas não se restringe a: médicos, enfermeiros, técnicos e auxiliares de enfermagem, cirurgiões-dentistas, nutricionistas e agentes comunitários de saúde (ACS).

**Comentário.** O trabalho em equipe, multiprofissional e transdisciplinar, é uma característica essencial da Estratégia de Saúde da Família (ESF) e da APS do SUS^91^. Estudos evidenciaram que os modelos de cuidado baseados na ESF, comparados com os modelos tradicionais de assistência na APS, estão associados a melhores indicadores de qualidade assistencial e a um maior acesso da população aos serviços de saúde, especialmente das pessoas com maior vulnerabilidade social^92–95^. O impacto positivo da participação de trabalhadores comunitários de saúde na APS foi também demonstrado na qualidade da assistência à DRC^96,97^.

**Recomendação 13**. As atividades da EPS devem estar alinhadas com a PNAB, com as ações de planejamento municipais vigentes, com um protocolo de DRC aprovado previamente no município e conter minimamente os seguintes temas: rastreamento de DRC, manejo de DRC na APS e critérios de encaminhamento para a AEA.

**Comentário**. Os profissionais responsáveis pela elaboração das ações de EPS devem considerar a PNAB, as rotinas de trabalho e a autonomia dos municípios na gestão da APS^36^. Sempre que possível, as atividades de EPS em DRC devem ser legitimadas pelo Plano Municipal de Saúde, pela Programação Anual de Saúde e estar incluídas no orçamento anual do exercício correspondente^98^. O protocolo municipal de rastreamento da DRC, detalhado na seção 3.3 deste documento (recomendações 3 a 10), deve ser aprovado previamente ao início das atividades de EPS para dar legitimidade ao conteúdo ensinado.

**Recomendação 14**. As ações de EPS no tema da DRC na APS devem: (1) ser realizadas no horário do expediente normal dos trabalhadores; (2) ser recorrentes e de curta duração; (3) ter objetivos e indicadores definidos; (4) utilizar metodologias de problematização; e (5) incluir momentos síncronos entre alunos e professores.

**Comentário**. O objetivo final da PNEPS deve ser a transformação das práticas de saúde por meio do desenvolvimento dos profissionais do SUS, aumentando a autonomia e a resolubilidade da APS^87^. Para alcançar esses resultados, as ações de EPS devem considerar os seguintes fatores: muitos trabalhadores das áreas da saúde possuem mais de um vínculo empregatício (especialmente a categoria dos médicos)^99–101^; existe alta rotatividade dos trabalhadores do SUS, sendo que esse fenômeno é ainda mais pronunciado na APS^102–104^; e, em geral, existe alta demanda assistencial nas unidades de saúde.

Portanto, é essencial que as atividades educativas aconteçam no horário de expediente normal de trabalho para aumentar a adesão dos profissionais de saúde; sejam recorrentes para que o conhecimento dos novos profissionais sobre as rotinas de trabalho esteja alinhado com o dos mais antigos; tenham conteúdo pragmático, objetivos claros e indicadores mensuráveis; apresentem tempo de duração que considere a demanda assistencial da unidade de saúde; e permitam o diálogo e o compartilhamento de ideias entre professores e alunos, uma vez que o grau de vivência no contexto local pode variar entre os profissionais envolvidos.

### Coordenação do cuidado integral em todos os estágios da DRC

**Recomendação 15**. Os governos dos três entes federativos devem unir esforços para garantir condições para que a APS seja a coordenadora do cuidado integral às pessoas com DRC em todos os estágios.

**Comentário**. As pessoas com as categorias de risco alto e muito alto de DRC e aquelas em TRS devem ser assistidas na atenção secundária e terciária à saúde, respectivamente^25–28^. Além disso, esses pacientes devem manter vínculo com a APS, considerando as seguintes necessidades específicas, mas não se restringindo a elas: vacinação, assistência em saúde bucal, dispensação de medicamentos essenciais, transporte sanitário e apoio na adesão a auxílios sociais^25,36^.

Comparados com a população geral, indivíduos com DRC possuem maior idade média, carga de doenças e vulnerabilidade social e, portanto, maiores demandas em saúde^105–107^. Ao longo do último século, as ciências da saúde, enquanto área de conhecimento, têm apresentado elevação exponencial de novas descobertas^108,109^. Junto com esse fenômeno, o número de especialidades médicas e não médicas aumentou, assim como a expectativa de vida e a prevalência de doenças crônicas^110,111^. Como consequência, a assistência às pessoas com multimorbidades se tornou um desafio para os sistemas de saúde^112,113^. Em que pese a legislação brasileira vigente, que é bem clara quanto ao papel da APS de ser a instância responsável pela coordenação do cuidado integral^25,36^, devem-se reconhecer as limitações de financiamento, a falta de recursos tecnológicos e a ausência de condições ideais para o desenvolvimento dos gestores públicos, especialmente no nível da APS^114–117^.

A APS deve coordenar uma cadeia de cuidados progressivos à saúde, que promova a integralidade e a resolução. O cuidado integral deve ser pautado pela longitudinalidade, assegurando o vínculo entre os profissionais da APS e os usuários ao longo do tempo, independentemente do estágio da DRC^25^. No caso da assistência a pacientes com DRC de alto e muito alto risco, a APS deve ser o centro da comunicação regional, reduzindo, assim, os riscos de iatrogenia decorrente da duplicidade de condutas e proporcionando a prestação de cuidados relacionados ou não com a DRC^118–120^.

### Gestão por indicadores

**Recomendação 16**. Sempre que possível, as ações de gestão da DRC na APS devem ser monitoradas por indicadores de qualidade com as seguintes características: relevância científica, objetividade, disponibilidade dos dados, custo de avaliação viável, reprodutibilidade e presença de metas para comparação (*benchmarks*).

**Comentário**. A utilização de indicadores é essencial na gestão pública, sobretudo em um cenário de aumento da carga de doenças, elevação dos gastos em saúde e recursos limitados^121,122^. A escolha do(s) indicador(es) deve ser cuidadosa e pautada em diversos pré-requisitos, entre os quais: relevância científica (o indicador deve ter associação com desfechos importantes como taxa de complicações e óbito); objetividade (a fórmula de cálculo deve ser claramente definida); viabilidade (os dados necessários para elaboração do indicador devem estar disponíveis e o custo para obtenção desses dados deve ser factível para a realidade local); reprodutibilidade (o indicador deve ser monitorado ao longo do tempo no mesmo local e passível de ser monitorado em outros locais); e presença de metas para comparação (o indicador deve ter uma meta definida com base em experiências anteriormente divulgadas, *benchmarks*)^123,124^.

Deve-se reconhecer que, na prática diária, principalmente em países de baixa e média renda, a obtenção de indicadores que possuem todas essas características não será possível em muitas ocasiões^125^. No contexto da assistência à DRC, existem indicadores que cumprem os requisitos descritos acima de modo razoável^126–130^. Além disso, esses indicadores podem ser utilizados separadamente para a gestão da DRC nos níveis primário e secundário (Quadro 3).

**Quadro 3.** Principais indicadores de qualidade da assistência à DRC na atenção primária e secundária (adaptado das refs. 126–130).

Nível de atenção	Indicadores
Primário	– % de pessoas com HA e/ou DM e ≥ 1 testagem de TFGe em 12 meses. – % de pessoas com HA e/ou DM e ≥ 1 testagem de RAC em 12 meses. – % de pessoas com HA e PA < 130 x 80 mmHg. – % de pessoas com DM e HbA1c < 7,0%. – % de pessoas com DRC em uso de IECA ou BRA, exceto se contraindicado. – % de pessoas com DRC em uso de inibidores de SGLT2, exceto se contraindicado. – % de pessoas com DRC com registro desse diagnóstico em prontuário médico.
Secundário	– % de pessoas com HA e PA < 130 x 80 mmHg. – % de pessoas com DM e HbA1c < 7,0%. – % de pessoas com DRC em uso de IECA ou BRA, exceto se contraindicado. – % de pessoas com DRC em uso de inibidores de SGLT2, exceto se contraindicado. – % de início de HD com FAV funcionante. – % de início de TRS sem internação. – % de início de TRS em diálise peritoneal. – % de encaminhamento para transplante renal preemptivo.

Abreviaturas – DRC: doença renal crônica; HA: hipertensão arterial; DM: diabetes mellitus; TFGe: taxa de filtração glomerular estimada; RAC: relação albumina/creatinina em amostra isolada de urina; PA: pressão arterial; HbA1c: hemoglobina glicada; IECA: inibidores da enzima conversora de angiotensina; BRA: bloqueadores do receptor de angiotensina; SGLT2: cotransportador sódio-glicose tipo 2; FAV: fístula arteriovenosa; TRS: terapia renal substitutiva.

Os principais indicadores de qualidade do cuidado em DRC na APS estão relacionados com: testagem de TFGe em pacientes de risco para DRC; testagem de RAC em pacientes de risco para DRC; controle de HA e DM; uso de medicações nefroprotetoras, exceto se contraindicado; registro do diagnóstico de DRC e encaminhamento para AEA de pacientes com risco de DRC alto ou muito alto. As fichas técnicas resumidas desses indicadores, com as metas propostas, estão descritas na Tabela 5 ^131–139^. A definição das metas para os indicadores selecionados é uma tarefa muitas vezes subjetiva, uma vez que devem ser considerados os recursos físicos, humanos e financeiros do local e as características individuais dos pacientes (idade, comorbidades, tolerância às medicações disponíveis, entre outras)^140^. Para além da definição dessa meta, os gestores públicos devem estabelecer planos de ação para melhorar o resultado dos indicadores^140,141^. Esses planos devem conter, de forma clara e objetiva, as atividades que serão realizadas, os profissionais responsáveis, os prazos de execução e os intervalos de monitoramento^141^.

**Tabela 5.** Ficha técnica dos indicadores de qualidade selecionados para monitorar a assistência à DRC na APS.

Indicador	Numerador	Denominador	Unidade	Meta	Variação	Ref.
Testagem de TFGe em pessoas com HA e/ou DM	Nº de adultos com HA e/ou DM e ≥ 1 dosagem de TFGe em 12 meses	Nº de adultos com HA e/ou DM	%	90%	Positiva*	29, 131, 132
Testagem de RAC em pessoas com HA e/ou DM	Nº de adultos com HA e/ou DM e ≥ 1 dosagem de RAC em 12 meses	Nº de adultos com HA e/ou DM	%	50%	Positiva	29, 131, 132
Controle de HA	Nº de adultos com HA e PA < 130 x 80 mmHg	Nº de adultos com HA	%	70%	Positiva	29, 133
Controle de DM	Nº de adultos com DM e HbA1c < 7,0%	Nº de adultos com DM	%	50%	Positiva	29, 134
Uso de IECA/BRA, exceto se contraindicado	Nº de adultos com DRC em uso de IECA ou BRA, exceto se contraindicado	Nº de adultos com DRC	%	80%	Positiva	29, 131
Uso de iSGLT2, exceto se contraindicado	Nº de adultos com DRC em uso de iSGLT2, exceto se contraindicado	Nº de adultos com DRC	%	50%	Positiva	30, 135–137
Reconhecimento da DRC	Nº de adultos com registro do diagnóstico de DRC em prontuário médico	Nº de adultos com DRC	%	40%	Positiva	29, 138, 139

Abreviaturas – TFGe: taxa de filtração glomerular estimada; HA: hipertensão arterial; DM: diabetes mellitus; IECA: inibidor da enzima conversora de angiotensina; BRA: bloqueador do receptor de angiotensina; iSGLT2: inibidores do cotransportador **sódio-glicose tipo 2**; Ref.: referência bibliográfica.

Nota – *Variação positiva do indicador: quanto maior o resultado, melhor.

## DISCUSSÃO

O presente artigo de consenso da SBN sintetiza um conjunto de 17 recomendações para apoiar a gestão da DRC na APS do SUS. Todas as recomendações foram sustentadas por legislações brasileiras e literatura científica especializada, incluindo portarias, diretrizes clínicas nacionais e consensos internacionais recentes. Além disso, o conteúdo foi amplamente discutido por membros da SBN e gestores das cinco regiões do Brasil, garantindo legitimidade e representatividade.

Em que pesem o subfinanciamento e a necessidade de modernização da gestão da saúde pública, especialmente em nível primário, a APS do SUS é reconhecida mundialmente por sua abrangência e resultados em ações de vacinação, combate a endemias, entre outras^142–144^. Entretanto, as mudanças epidemiológicas e demográficas que o Brasil atravessa adicionaram demandas em saúde desafiadoras^1,6,145,146^. Entre elas, destacam-se as DCNT, nas quais a DRC está fortemente inserida. Comparada ao DM, às doenças cardiovasculares, às doenças respiratórias crônicas e às neoplasias malignas, a DRC é a condição que tem apresentado o maior aumento de prevalência e os principais impactos na redução da expectativa de vida e nos gastos em saúde^147^.

O gasto *per capita* em saúde no Brasil é baixo quando comparado com aquele de países de alta renda. Além disso, somente 50% do montante desses recursos são consumidos pela assistência pública, da qual dependem exclusivamente pouco mais de 75% dos brasileiros^148,149^. Dentre os entes federativos (União, estados e municípios), os municípios são aqueles que têm a maior participação nas despesas em saúde, o que consome, em grande parte, os seus orçamentos anuais^41,42,150^.

A ESF é um modelo de cuidado consagrado, tanto em razão do melhor desempenho em indicadores assistenciais quanto devido à melhor percepção de qualidade pelos usuários, quando comparada ao modelo de unidades básicas de saúde tradicionais^92–95,151^. A cobertura de ESF é desigual nas regiões do Brasil e, dentro dos estados, observa-se também grande heterogeneidade^151,152^. Além disso, é preocupante a estagnação/redução da cobertura de agentes comunitários de saúde, categoria profissional que tem a maior proximidade com a população dentre todas as outras^153,154^.

A implementação das recomendações depende da articulação entre as instâncias de pactuação e gestão do SUS, tais como Secretarias Municipais e Estaduais de Saúde e as Comissões Intergestores Regionais (CIR), Bipartites (CIB) e Tripartite (CIT), responsáveis por definir fluxos assistenciais e incorporar a DRC nas agendas interfederativas^36,88,155^. Saliente-se que experiências nas cinco regiões do Brasil mostram que o tema da DRC tem sido pautado de forma a seguir o rito do SUS, ou seja, por meio de protocolos municipais e estaduais, notas técnicas, deliberações de CIB, entre outros^156–162^. Em acordo, recentemente, a Organização Mundial da Saúde incluiu a DRC na lista de condições crônicas não transmissíveis prioritárias para ações de financiamento, monitoramento, educação, prevenção, colaboração multissetorial e regional, entre outras^163^.

No tocante às ações de prevenção secundária, destaca-se que o rastreamento com creatinina e RAC é altamente custo-efetivo^13–16,164^, especialmente quando associado ao uso oportuno de medicamentos nefroprotetores disponíveis no SUS, como IECA, BRA e inibidores de SGLT2^26,70^. Indivíduos que realizaram pelo menos uma testagem de RAC nos últimos 12 meses apresentam maior chance de estarem utilizando nefroprotetores e de apresentarem controle de comorbidades, como HA, mesmo após ajuste por diversas variáveis confundidoras^81^. Entretanto, foram demonstradas baixa testagem de RAC e subutilização de IECA, BRA e inibidores de SGLT2 em diversos serviços do Brasil^29,30^, reforçando a importância das recomendações contidas neste consenso. De acordo com a diretriz vigente, a prescrição de inibidores de SGLT2 no SUS requer o preenchimento de um laudo de solicitação de medicamentos do componente especializado (LME), assim como a elaboração de um relatório médico e a apresentação de exames específicos^70^. Para amenizar, ao menos em parte, essa situação, que pode constituir uma barreira de acesso, alguns serviços têm adotado versões digitais da LME. O consenso também aborda propostas para territórios remotos e populações vulneráveis, sugerindo o uso de testes no local de atendimento (*point-of-care*) para creatinina e RAC. Essa abordagem é respaldada pela diretriz KDIGO 2024^28^ e mostrou-se eficaz em países de diferentes níveis de renda^76–79^, como comentado anteriormente.

Outro desafio relevante é a distribuição desigual de nefrologistas no país, impossibilitando o encaminhamento universal de todos os casos classificados como de risco alto ou muito alto no mapa de calor da DRC^28,37^. Modelos de matriciamento envolvendo nefrologistas têm demonstrado impacto positivo na qualificação das condutas, redução de encaminhamentos desnecessários e maior adesão ao rastreamento de DRC^86–90^. De fato, o matriciamento é amplamente recomendado em legislações e diretrizes do SUS e é fundamental para ampliar a resolubilidade da APS, mas carece ainda de políticas públicas de incentivo à fixação de nefrologistas em áreas remotas^24–26^.

A telessaúde emerge como ferramenta estratégica para ampliar o acesso, reduzir desigualdades regionais e integrar equipes de saúde, conforme já demonstrado em estudos nacionais e internacionais^118–120^. Teleinterconsultas, teleducação e telediagnóstico podem apoiar tanto a APS quanto a AEA. Da mesma forma, tecnologias emergentes baseadas em inteligência artificial, incluindo algoritmos de apoio à decisão clínica e predição de risco renal, tendem a se tornar parte importante da prática nefrológica nos próximos anos^108–113^, especialmente à medida que prontuários eletrônicos se expandem no SUS. Entretanto, persistem desafios estruturais, como a informatização incompleta das unidades básicas de saúde e a disponibilidade limitada de internet de banda larga em áreas rurais e municípios de pequeno porte.

Reconhece-se que a efetivação plena das recomendações depende da capacidade da União e dos estados de apoiar técnica e financeiramente a APS^165,166^ e integrá-la mais eficientemente à AEA e aos serviços hospitalares e de TRS. Entretanto, é seguro e recomendado que a APS não referencie mais de 95% dos indivíduos sob risco ou com DRC, reforçando seu papel central no enfrentamento da DRC e de outras DCNT^25,36^. A gestão orientada por indicadores, conforme proposta neste documento, representa uma ferramenta robusta para monitorar resultados, identificar fragilidades, corrigir rotas e promover melhoria contínua, mesmo em contextos de restrição orçamentária^121–130^.

## CONCLUSÃO

As recomendações apresentadas neste consenso constituem um marco estratégico para a gestão da DRC na APS do SUS. Elas alinham evidências científicas, políticas públicas, instrumentos de gestão e experiências regionais, oferecendo aos gestores um guia prático e factível para melhorar o diagnóstico precoce, ampliar o acesso a terapias nefroprotetoras, fortalecer a coordenação do cuidado e reduzir a progressão da doença. A consolidação dessas ações requer pactuação interfederativa, investimentos sustentados, qualificação profissional recorrente e incorporação progressiva de tecnologias e inovações.

## Data Availability

Este manuscrito foi elaborado com base na revisão, síntese e interpretação da literatura científica, da legislação e de documentos técnicos. Não foram gerados conjuntos de dados originais.
